# *Saccharomyces cerevisiae* Δ9-desaturase Ole1 forms a supercomplex with Slc1 and Dga1

**DOI:** 10.1016/j.jbc.2023.104882

**Published:** 2023-06-01

**Authors:** Brianna L. Greenwood, Zijun Luo, Tareq Ahmed, Daniel Huang, David T. Stuart

**Affiliations:** Department of Biochemistry, University of Alberta, Edmonton, Alberta, Canada

**Keywords:** acyltransferase, Δ9 desaturase, Ole1, Dga1, Slc1, *S. cerevisiae*, triacylglycerol

## Abstract

Biosynthesis of the various lipid species that compose cellular membranes and lipid droplets depends on the activity of multiple enzymes functioning in coordinated pathways. The flux of intermediates through lipid biosynthetic pathways is regulated to respond to nutritional and environmental demands placed on the cell necessitating that there be flexibility in pathway activity and organization. This flexibility can in part be achieved through the organization of enzymes into metabolon supercomplexes. However, the composition and organization of such supercomplexes remain unclear. Here, we identified protein–protein interactions between acyltransferases Sct1, Gpt2, Slc1, Dga1, and the Δ9 acyl-CoA desaturase Ole1 in *Saccharomyces cerevisiae*. We further determined that a subset of these acyltransferases interact with each other independent of Ole1. We show that truncated versions of Dga1 lacking the carboxyl-terminal 20 amino acid residues are nonfunctional and unable to bind Ole1. Furthermore, charged-to-alanine scanning mutagenesis revealed that a cluster of charged residues near the carboxyl terminus was required for the interaction with Ole1. Mutation of these charged residues disrupted the interaction between Dga1 and Ole1 but allowed Dga1 to retain catalytic activity and to induce lipid droplet formation. These data support the formation of a complex of acyltransferases involved in lipid biosynthesis that interacts with Ole1, the sole acyl-CoA desaturase in *S. cerevisiae*, that can channel unsaturated acyl chains toward phospholipid or triacylglycerol synthesis. This desaturasome complex may provide the architecture that allows for the necessary flux of de novo–synthesized unsaturated acyl-CoA to phospholipid or triacylglycerol synthesis as demanded by cellular requirements.

The metabolic pathways responsible for the synthesis and assembly of glycerophospholipid species play a key role in establishing cellular membrane structure and controlling energy homeostasis (1). The synthesis of glycerophospholipid species is a complex process starting with the initial carboxylation of acetyl-CoA to malonyl-CoA by acetyl-CoA carboxylase. Fatty acyl-CoA synthetase (FAS) then catalyzes condensation of acetyl-CoA and malonyl-CoA into saturated acyl-chains with 16 to 18 carbons being the most common (2). In *Saccharomyces cerevisiae*, a high proportion of acyl-CoA undergoes monounsaturation catalyzed by the stearoyl-CoA desaturase Ole1 in the endoplasmic reticulum (ER) (3) (Fig. 1*A*). Subsequently, saturated and unsaturated acyl-CoA are assembled into a diverse family of glycerophospholipids whose composition, chain length, and degree of unsaturation influence membrane properties and structure (4).

**Figure 1:**
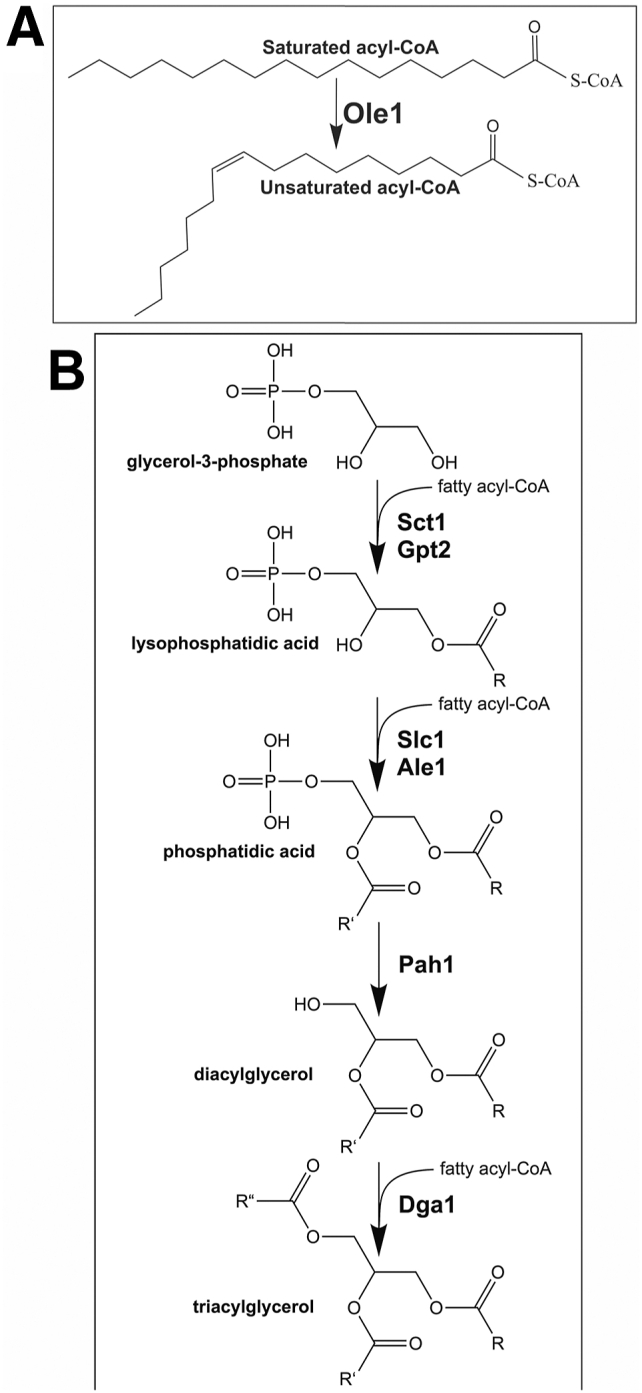
**The pathway to triacylglycerol synthesis in *S. cerevisiae*.***A*, Δ9 acyl-CoA desaturase Ole1 is required to generate unsaturated acyl-CoA species. *B*, the pathway to triacylglycerol in *S. cerevisiae*. Enzymes catalyzing the consecutive reactions are shown in *bold*. Acyltransferases mediate the synthesis of lysophosphatidic acid and phosphatidic acid (PA). Dephosphorylation of PA yields diacylglycerol that acts as a substrate for Dgat activities to synthesize triacylglycerol.

The assembly of glycerophospholipids is initiated by glycerophosphate acyltransferases encoded by *GPT2* and *SCT1* that form lysophosphatidic acid (LPA) by adding an acyl chain to glycerol 3-phosphate (5) (Fig. 1*B*). This is followed by lysophosphatidic acid acyltransferases encoded by *SLC1* and *ALE1* that add an acyl chain to the *sn*-2 position of LPA to form phosphatidic acid (PA) (6, 7) (Fig. 1*B*). Slc1 and Ale1 are both found in the ER membrane but only Slc1 localizes to lipid droplets (LDs) (8). Slc1 and Ale1 have an apparent strong preference for C18:1-CoA, and this specificity is reflected in the high proportion of phospholipid and neutral lipid species that display C18:1 at the *sn*-2 position (9, 10). The PA formed in these reactions can be further modified to CDP-DAG and acts as precursor to the primary membrane phospholipids (11). PA can also be subjected to dephosphorylation by phosphatase Pah1 to yield diacylglycerol (DAG) that can act as substrate for diacylglycerol acyltransferases (DGATs) Dga1 and Lro1 to form triacylglycerol (TAG) (12, 13, 14) (Fig. 1*B*). Dga1 catalyzes the acylation of DAG with acyl-CoA for the synthesis of TAG and is responsible for a large fraction of the TAG synthesized in *S. cerevisiae* (13). Lro1 also forms TAG but transfers an acyl-chain from an existing phospholipid to DAG rather than utilizing *de novo* synthesized acyl-CoA (14). Dga1 displays localization both in the ER membrane and LDs, whereas Lro1 is confined the ER membranes (15). Fatty acyl chains can also be also stored as sterol esters in reactions catalyzed by Are1 and Are2 (16).

Fatty acid biosynthetic pathways can of necessity respond to rapidly changing physiological demands and environmental conditions, yet little is known regarding how they are spatially organized and coordinated to provide this responsiveness. It has been proposed that pathway efficiencies can be optimized through physical interaction between enzymes that act sequentially in a pathway, thus organizing them into metabolons (17). Such organization could promote channeling of intermediates to minimize their diffusion into the cytoplasm or surrounding membrane environments (18). This could provide for precision in the regulation of the flux of intermediates toward specific products through association and dissociation of individual subunits within an enzyme supercomplex (19). There is evidence for the existence of enzyme supercomplexes for lipid biosynthesis assembled through the interaction of FAS with ATP citrate lyase and acetyl-CoA carboxylase to provide acetyl-CoA and malonyl-CoA substrates and malic enzyme, pyruvate carboxylase, and malate dehydrogenase that provide the NADPH required for FAS to synthesize acyl-chains (20). A soluble TAG synthesizing complex containing LPA acyltransferase, PA phosphatase, DGAT, acyl-acyl carrier protein synthetase, and acyl-acyl carrier protein activities was identified in the oleaginous yeast *R. glutinis*. This complex is stabilized by protein–protein interactions and can incorporate acyl-CoA as well as free fatty acids into TAG and its biosynthetic intermediates (21).

Physical interactions have also been demonstrated among acyltransferases, and there is an implication that these interactions direct the synthesis of distinct TAG species in several plant models (22, 23, 24). Similarly, transfected DGAT2 and monoacylglycerol transferase-2 display colocalization and physical interaction in a human cell model (25). It is likely that other proteins interact with DGAT2 in this human cell model since cross-linking studies using both purified membrane fractions and whole cells indicate that DGAT2 exists in a complex that migrates at approximately 650 kDa on polyacrylamide gels, consistent with it being a component of an enzyme supercomplex (25). Thus, lipid biosynthetic pathways assembled into enzyme supercomplexes may be wide-spread, but the spatial organization of these complexes and the protein–protein interactions that scaffold them together remain uncharacterized.

Acyl-CoA desaturases play a key role in regulating membrane and storage lipid composition as they introduce double bonds into acyl chains that influence the chemical and physical properties of membrane phospholipids (26). Indeed, in *S. cerevisiae* 75 to 90% of fatty acids are unsaturated, and the stearoyl-CoA desaturase encoded by *OLE1* is essential for viability (3, 27). Inactivation of Ole1 leads to a reduction in TAG abundance in *S. cerevisiae* and an increase in saturated acyl chains in both TAG and phospholipid species (28).The mouse stearoyl-CoA desaturase SCD1 is not essential, but SCD1^−/−^ mice have significantly reduced fat stores (29). Similarly, SCD1 has a role in determining TAG abundance and phospholipid composition in *Caenorhabditis elegans* (30). These observations suggest that stearoyl-CoA desaturase may play a key role in the biosynthesis of membrane phospholipids and TAG that extends beyond its capacity to introduce double bonds into acyl-chains.

Numerous acyl-CoA desaturases including Ole1 have been demonstrated to function as homodimers, and some acyl-CoA desaturases like *Arabidopsis thaliana* FAD2 and FAD3 form a heterodimer complex to channel oleate to linolenate without allowing a linoleoyl intermediate to be released (31). Owing to the importance of unsaturated acyl-chains in determining the physical properties of membrane phospholipids and the role of stearoyl-CoA desaturase in supplying unsaturated acyl-chains as substrate for acyltransferases, an important question then is: Do acyl-CoA desaturases form physical complexes with acyltransferases to channel unsaturated acyl chains to specified phospholipid or neutral lipid species? In cultured HeLa cells, overexpressed acyl-CoA desaturase SCD1 and acyltransferase DGAT2 colocalize and can be coimmunoprecipitated, consistent with a physical interaction between the two enzymes (32). Additionally, biochemical analysis of microsomal membranes suggests there is channeling of lipid species between oleoylglycerophosphocholine desaturase and monoacylglycerophosphocholine acyltransferase in *Pisum sativum* without intermediate species equilibrating with the bulk membrane pool of phospholipid (33).

In contrast to the extensive biochemical evidence supporting complex formation among enzymes that catalyze lipid biosynthesis, there is little structural data to provide information around the protein domains and sequences involved in making protein–protein interactions among these enzymes. The crystal structure of human stearoyl-CoA desaturase SCD1 has been solved, but this has not revealed sites of interaction with other enzymes (34).

The structure of human acyltransferase DGAT1 revealed that the dimer interface includes transmembrane helices and contacts occurring within the lipid bilayer (35). Although no structural data are available for DGAT2, domains implicated in ER membrane targeting and LD binding have been elucidated by deletion analysis. However, these studies have yet to reveal how DGAT2 interacts with the triglyceride synthesizing supercomplex it was found to be part of and whether the membrane context influences these interactions (25, 36, 37).

Here we have employed *S. cerevisiae* to investigate the hypothesis that interactions exist among stearoyl-CoA desaturase and acyltransferases responsible for phospholipid and TAG synthesis. We have identified physical interactions between acyltransferases Slc1 and Dga1 and between both acyltransferases and desaturase Ole1. The interaction between Dga1 and Ole1 requires the carboxyl terminus of Dga1 required for enzymatic activity. We refer to this desaturase-acyltransferase biosynthetic complex as a desaturasome. Further, we demonstrate physical interaction between Slc1 and Dga1 independent of Ole1. These findings are consistent with the contention that protein–protein interactions among these enzymes can act to channel acyl-CoA toward specific glycerolipid species and may be one mechanism by which control can be imposed over membrane composition and physical properties as well as storage lipid composition.

## Results

### Δ9 desaturase Ole1 interacts with acyltransferases Sct1, Gpt2, Slc1, and Dga1

**Figure 2:**
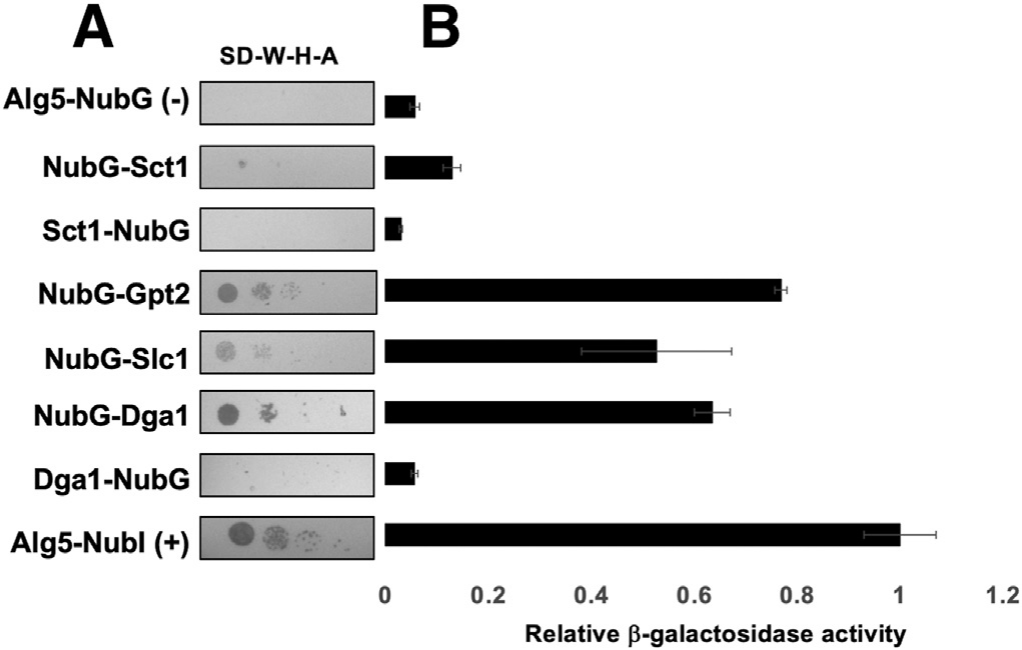
**Δ9 acyl-CoA desaturase Ole1 interacts with acyltransferases.***A*, integrated membrane yeast 2-hybrid assay using a yeast strain expressing an endogenously tagged Ole1-Myc-C_ub_-LexA-VP16 as “bait” and the indicated NubG fusions as “prey”. Growth on selective medium plates is representative of three separate experiments (n = 3). Synthetic defined medium lacking tryptophan, histidine, and adenine (SD-W-H-A) was used for the growth test. *B*, β-galactosidase activity in Miller units assayed from cell extracts. Each column represents the mean of three biological replicates (n = 3). Activities were normalized to the positive control (*pALG5-NubI*). Error bars reflect standard deviation.

Ole1 is a central component in the *de novo* synthesis of phospholipids and TAG by acting as the source of unsaturated acyl-CoA that can be distributed to acyltransferases. We applied a split ubiquitin strategy to test for *in vivo* interactions between Ole1 and acyltransferase enzymes that have roles in glycerolipid biosynthesis. Ole1 displays 60% similarity to the rat stearoyl-CoA desaturase (3). The structure of the rat enzyme has been solved, and the topology of stearoyl-CoA desaturase in rat orients both the amino and carboxyl termini toward the cytosol, with the active site also positioned facing the cytosol (38). On this basis, we introduced the Cub-LexA-VP16 tag to the carboxyl end of Ole1. When a plasmid borne Ole1-Cub-LexA-VP16 fusion was introduced into the reporter strain NMY51, the construct induced activation of the reporter genes independent of any binding partner. We hypothesized that the strong autoactivation might have occurred owing to degradation of the tagged version of Ole1 since cells are sensitive to Ole1 dosage (39). This limitation was overcome by employing an integrated membrane yeast two-hybrid assay by introducing a DNA sequence encoding a Myc-Cub-LexA-VP16 tag into the endogenous chromosomal *OLE1* in the reporter strain NMY51. This Ole1 “bait” strain (YBG1) was subsequently transformed with negative and positive control vectors (pALG5-NubG, pALG5-NubI), in addition to plasmids harboring the coding sequences for acyltransferases *SCT1*, *GPT2*, *SLC1*, and *DGA1* fused to the amino terminus of ubiquitin (Fig. 2*A*). The *OLE1* bait strain harboring positive control pALG5-NubI displayed growth as expected on selective medium, whereas the negative control pALG5-NubG did not activate the *HIS3* and *ADE2* reporter genes, and no growth could be observed on the selective medium (Fig. 2*A*). Expression of NubG-Gpt2, NubG-Slc1, and NubG-Dga1 fusions activated the *HIS3* and *ADE2* reporter genes, permitting growth under selection conditions (Fig. 2*A*). The NubG-Sct1 fusion displayed weaker growth on the selective plates but was detectable above the negative control. In contrast, when the NubG tag was fused to the carboxyl terminus of Sct1 (Sct1-NubG) and the carboxyl terminus of Dga1 (Dga1-NubG), no growth was observed on the selective medium (Fig. 2*A*). Expression of the LacZ reporter gene was consistent with the extent of cell growth observable on the selective medium showing that the NubG-Sct1, NubG-Gpt2, NubG-Slc1, and NubG-Dga1 fusions all induced a significant increase in expression of β-galactosidase activity over the negative control Alg5-NubG (Fig. 2*B*). Expression of NubG-Sct1 increased activity twice that of the negative control (*p* = 0.003). NubG-Slc1 expression in YBG1 increased β-galactosidase activity 9-fold over the negative control (*p* = 0.006), while NubG-Dga1 expression in YBG1 increased activity 11-fold (*p* << 0.005). Expression of the NubG-tagged acyl-transferases in the NYM51 strain was confirmed by Western blot analysis of whole cell lysates (Fig. S1). These observations provide the first demonstration that acyltransferases Sct1, Gpt2, Slc1, and Dga1 interact with Δ9 desaturase Ole1 *in vivo*.

### Slc1 interacts with Dga1

**Figure 3:**
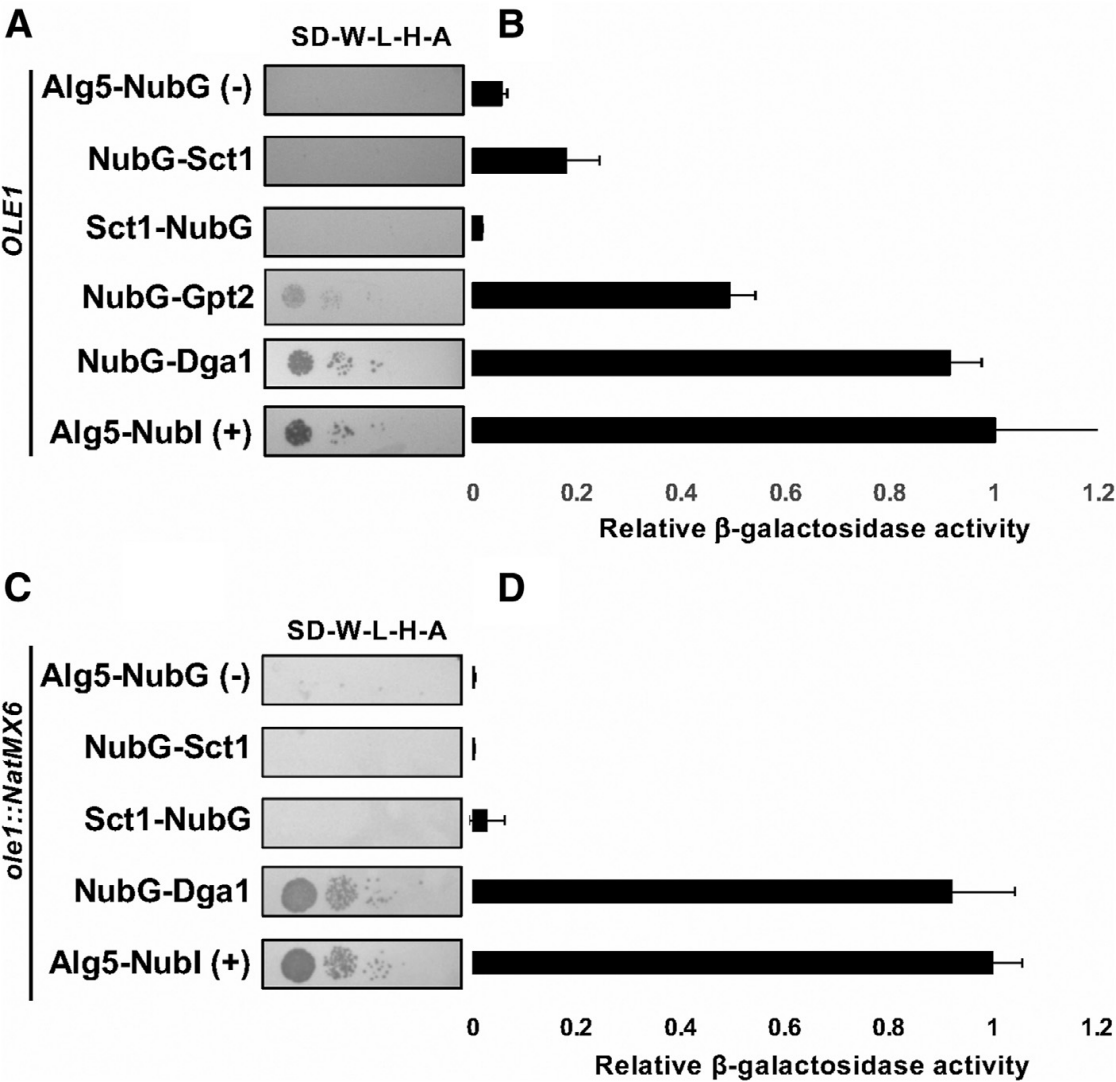
**Acyltransferase Slc1 interacts with Dga1.***A*, integrated membrane yeast two-hybrid assay using a yeast strain expressing Slc1-C_ub_-LexA-VP16 as bait and the indicated NubG fusions as “prey”. Growth on selective medium plates is representative of three separate experiments (n = 3). Synthetic defined medium lacking tryptophan, leucine, histidine, and adenine (SD-W-L-H-A) was used for the growth test. *B*, β-galactosidase activity in Miller Units assayed from cell extracts. Each column represents the mean of three biological replicates (n = 3). Activities were normalized to the positive control (*pALG5-NubI*). Error bars reflect standard deviation. *C*, integrated membrane yeast 2-hybrid assay using a *Δole1* strain expressing Slc1-C_ub_-LexA-VP16 as bait and the indicated NubG fusions as “prey”. Growth on selective medium plates (SD-W-L-H-A) supplemented with 0.5% Tween-80 is representative of three separate experiments (n = 3). *D*, β-galactosidase activity in Miller units assayed from *Δole1* cell extracts. Each column represents the mean of three biological replicates (n = 3). Activities were normalized to the positive control (*pALG5-NubI*). Error bars reflect standard deviation.

Following the observation that Ole1 interacts with acyltransferases Sct1, Gpt2, Slc1, and Dga1, we were interested in determining whether the acyltransferases interacted with one another as an acyltransferase interactome has been demonstrated in *Linum usitatissimum* (22). A reporter strain harboring *SLC1*-Cub-LexA-VP16 as “bait” (YBG2) was transformed with negative and positive control vectors (p*ALG5*-NubG, p*ALG5*-NubI), in addition to acyltransferase fusions, pNubG-*SCT1*, p*SCT1*-NubG, pNubG-*GPT2*, and pNubG-*DGA1*. The Slc1 bait strain containing the positive control prey plasmid p*ALG5*-NubI displayed growth on selective medium, whereas the negative control p*ALG5*-NubG displayed no growth as expected (Fig. 3*A*). Both of the NubG-Gpt2 and NubG-Dga1 fusions in combination with the *SLC1*-Cub-LexA-VP16 fusion activated the *HIS3* and *ADE2* reporter genes allowing growth on the selective medium (Fig. 3*A*). Neither of the NubG-*SCT1* or *SCT1*-NubG fusions were able to induce robust growth when paired with the *SLC1*-Cub-LexA-VP16 fusion (Fig. 3*A*). However, when interaction stringency was investigated with a β-galactosidase activity assay, the NubG-*SCT1* fusion in combination with *SLC1*-Cub-LexA-VP16 exhibited three times greater β-galactosidase activity than the negative control (*p* = 0.008), while expression of NubG-Gpt2 and NubG-Dga1 increased β-galactosidase activity almost 10-fold and 17-fold, respectively, over Alg5-NubG (*p* << 0.005). These data indicate that the *S. cerevisiae* acyltransferases, Sct1, Gpt2, and Dga1, interact with Slc1 as well as with Ole1 *in vivo*.

To gain further insight into the organization of the acyltransferase–desaturase interactions, we extended this investigation to test whether the Slc1 and Dga1 could interact with one-another independent of Ole1. The *OLE1* coding sequence was deleted from the reporter strain NMY51 to yield *ole1*::NatMX6 (YGB3). This strain was maintained on medium supplemented with unsaturated fatty acids to support viability, and plasmids encoding *SLC1*-Cub-LexA-VP16 and NubG-*DGA1* were installed in the strain. The *ole1*-deletion mutant displayed growth on the selective medium indicating that Slc1 and Dga1 fusions were able to interact with one another in the absence of Ole1 (Fig. 3, *C* and *D*). Thus, while Slc1 and Dga1 both interact with Ole1, they also interact with one another, and Ole1 is not required as a scaffold for this interaction.

### The carboxyl terminus of Dga1 is required for its interaction with Ole1

**Figure 4:**
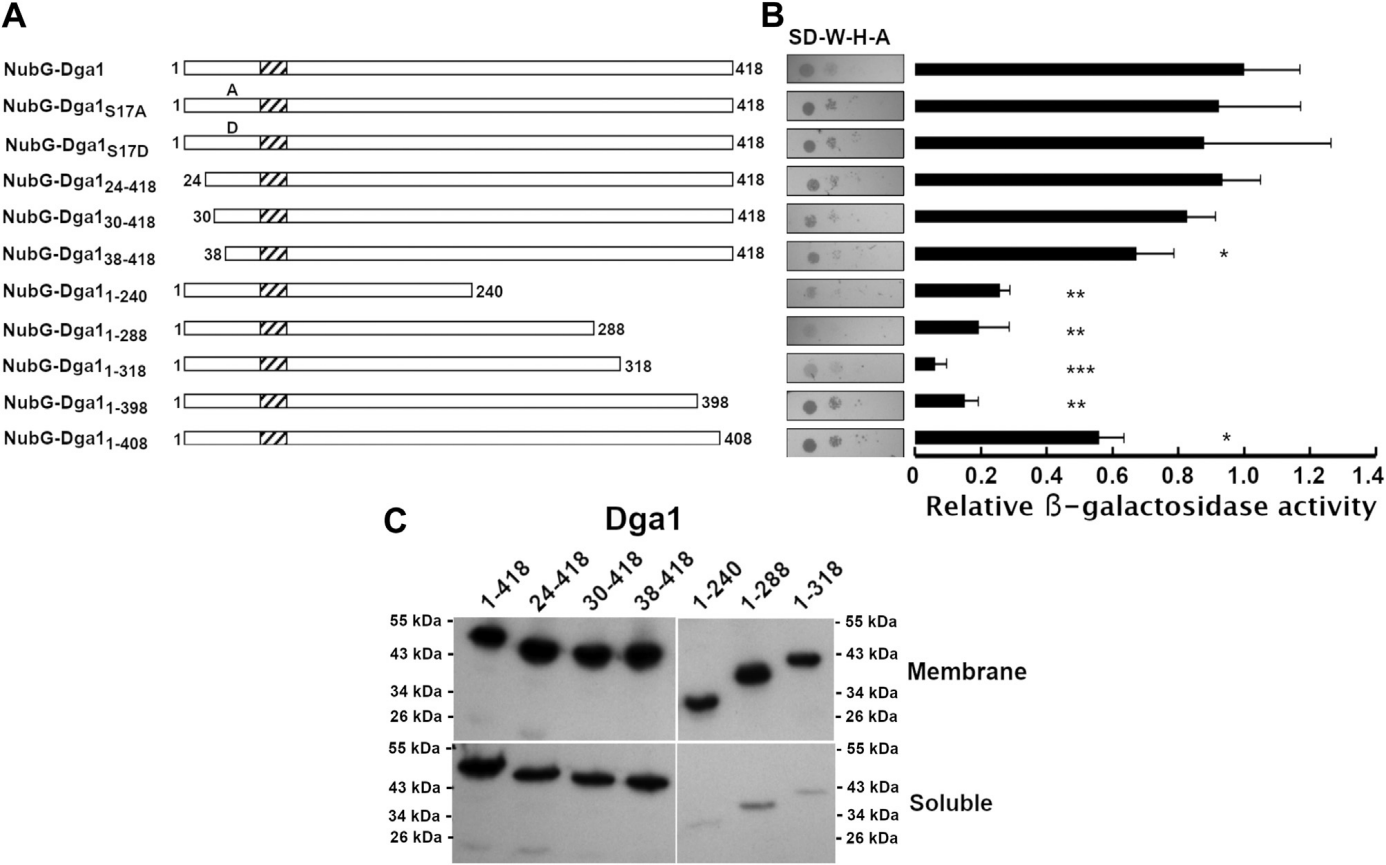
**Truncation of the Dga1 carboxyl terminus disrupts the interaction with Ole1.***A*, schematic map of the Dga1 truncations tested, the *dashed box* represents the predicted transmembrane domain. *B*, integrated membrane yeast two-hybrid assay using a yeast strain expressing Ole1-Myc-C_ub_-LexA-VP16 as “bait” and the indicated constructs fused to NubG as “prey”. Growth on selective medium plates is representative of three independent experiments. Synthetic defined medium lacking tryptophan, histidine, and adenine (SD-W-H-A) was used for the growth test. Bars represent β-galactosidase assay of a yeast strain expressing Ole1-Myc-C_ub_-LexA-VP16 and the indicated NubG-Dga1 variants. Each column is representative of three biological replicates (n = 3). Miller units were normalized to the NubG-full-length Dga1 fusion. *Asterisks* (∗) denote significantly different *LacZ* activity from NubG-Dga1 by Student’s *t* test, (∗*p* < 0.05, ∗∗*p* < 0.01, ∗∗∗ *p* < 0.001). Error bars reflect standard deviation. *C*, Western blot analysis of the proportion of Dga1 found in the membrane (*upper panel*) and soluble (*lower panel*) fractions. The Dga1 variants are indicated at the *top* of each lane. The migration of molecular weight markers (kDa) is indicated to the side of each panel.

Protein–protein interactions between integral membrane proteins can be complex relationships influenced by both the protein partners and the lipid bilayer environment and composition. To further investigate our novel observation that Dga1 interacts with Ole1 in the ER membrane, we performed a mutagenic analysis of Dga1 to determine if a specific domain of Dga1 responsible for maintaining the interaction with Ole1 could be identified. Topological analysis of Dga1 indicates that the amino and carboxyl termini are oriented to the cytoplasmic side of the ER membrane (40). We initiated a mutagenic analysis by generating truncations of the amino and carboxyl termini of Dga1 and fusing the truncations in frame with NubG to determine if they were capable of interacting with the integrated Ole1-Cub-LexA-VP16 bait in YBG1 (Fig. 4*A*). The amino-terminal 36 amino acids residues of Dga1 have been implicated in regulation of the enzyme's activity, and within this segment of the protein, the residue Ser^17^ is subject to modification by phosphorylation (41, 42). To determine if Ser^17^ phosphorylation influences Dga1 interaction with Ole1, the Ser^17^ residue was mutated to either nonphosphorylatable Ala^17^ or the phosphomimetic Asp^17^. Neither change had any detectable effect on the Ole1–Dga1 interaction as measured by growth of the cells on selective medium or quantitative β-galactosidase activity (Fig. 4*B*). Consistent with this observation, deletion of Dga1 amino acids 1 to 29 had no significant effect on interaction of Dga1 with Ole1 as evaluated by growth of the strains on selective medium and quantitative β-galactosidase activity (Fig. 4*B*). Further deletion of amino acids 1 to 37 (NubG-Dga1_38–418_) caused a significant reduction in β-galactosidase activity to about 67% of the full-length Dga1 (*p* = 0.015) (Fig. 4*B*).

A truncation that deleted amino acids 319 to 418, predicted to form a carboxyl-terminal cytoplasmic domain (NubG-Dga1_1–318_), resulted in a loss of interaction with Ole1 based upon a reduction of growth observed on selective medium and the near complete elimination of β-galactosidase activity (Fig. 4*B*). Further deletions from the carboxy terminus (NubG-Dga1_1–240_, NubG-Dga1_1–288_) showed no further reduction in the interaction based on β-galactosidase assay (Fig. 4*B*). A shorter truncation of the last 20 amino acids of the carboxy-terminal cytoplasmic domain of Dga1 (NubG-Dga1_1–398_) also abolished the interaction with Ole1, demonstrated by a loss of growth on selective medium and a decrease in β-galactosidase activity (Fig. 4*B*). A Dga1 variant lacking the final ten amino acids (NubG-Dga1_1–408_) supported more extensive growth on selective medium and increased β-galactosidase activity consistent with this truncated variant displaying interaction with Ole1 (Fig. 4*B*). NubG-Dga1_1–408_ induced ∼200% more β-galactosidase activity that did NubG-Dga1_1–398_, suggesting that residues 398 to 418 might play an important role in the interaction of Dga1 and Ole1 (Fig. 4*B*).

The ability of Dga1 truncations to bind Ole1 and activate the reporter genes in the two-hybrid assay are dependent upon their expression and localization to the ER membrane. Expression and localization of the Dga1 variants was tested by Western blot analysis and subcellular fractionation. Probing whole cell lysates with anti-HA antibodies revealed that the NubG fusion to full-length Dga1 and all of the amino- and carboxyl-terminal truncations from Dga1_24–418_ to Dga1_1–318_ were expressed (Fig. S1*B*). To confirm that the Dga1 variants were being correctly localized in membranes, whole cell extracts were subjected to centrifugation at 104,000*g* for 1 h, and samples of the soluble fraction and pelleted membrane fraction were assayed for Dga1 by Western blot. Comparison of Western blots indicates that over 60% of Dga1_24–418_, Dga1_30–418_, and Dga1_38–418_ were present in the pellet fraction consistent with membrane localization (Fig. 4*C*). The Dga1 carboxy-terminal truncations Dga1_1–240_, Dga1_1–288_, and Dga1_1–318_ all display predominant localization to the membrane fraction (Fig. 4*C*).

### Charged residues at the carboxyl terminus of Dga1 are important for the Ole1–Dga1 interaction

**Figure 5:**
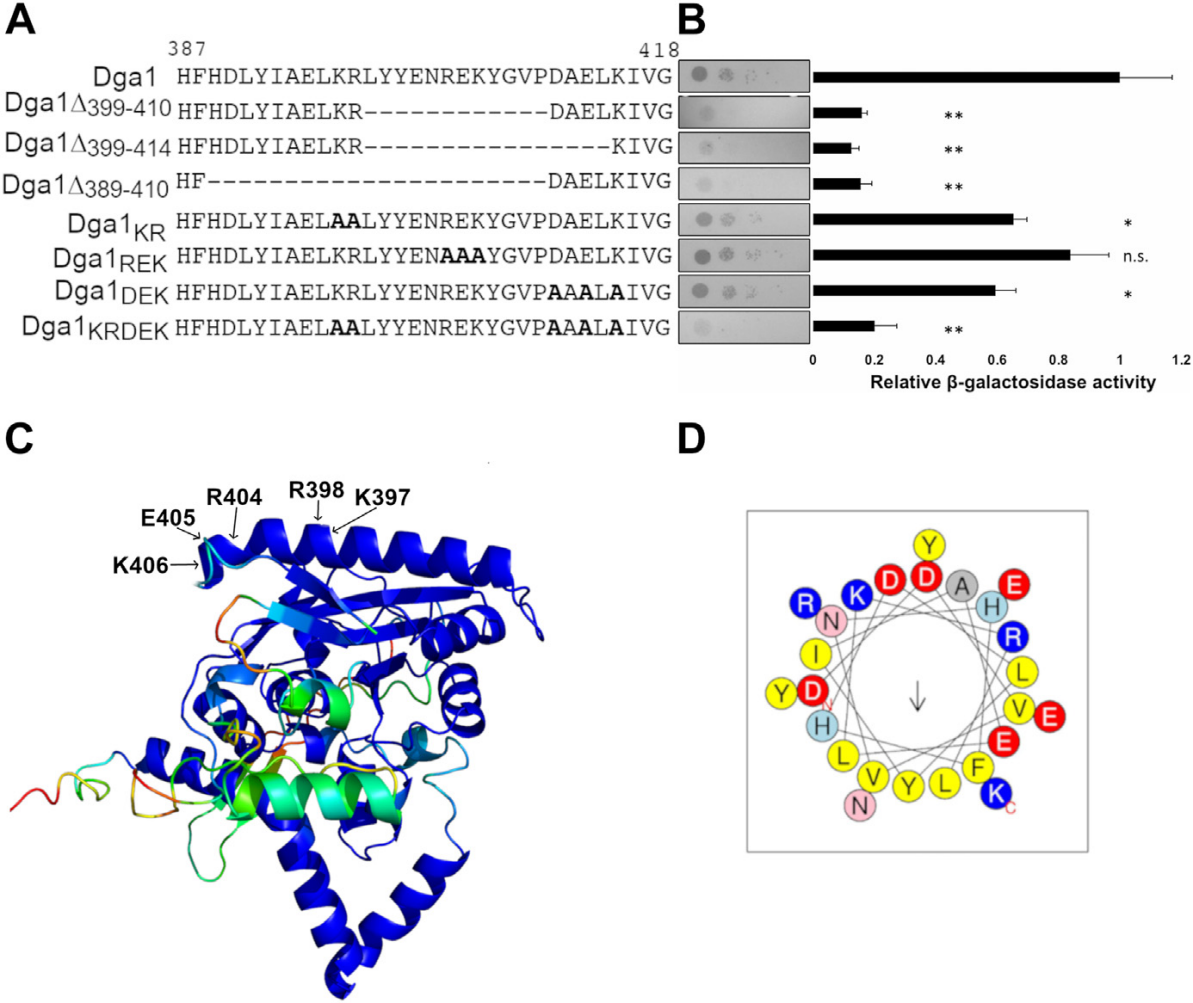
**Charged residues at the carboxyl terminus of Dga1 are important for interaction between Ole1 and Dga1.***A*, sequences of carboxyl-terminal Dga1 mutants. *Dashed lines* indicate deleted sequences. *Bold* A indicates replacement of a charged residue with alanine. *B*, integrated membrane yeast two-hybrid assay using a yeast strain expressing Ole1-Myc-C_ub_-LexA-VP16 and the indicated NubG-Dga1 variants. Growth on selective medium plates is representative of three independent experiments. *Filled bars* represent β-galactosidase activity in Miller units from extracts of the yeast strain expressing Ole1-Myc-C_ub_-LexA-VP16 and the indicated NubG-Dga1 fusions. Each column is representative of three biological replicates (n = 3). Miller units were normalized to the NubG-full-length Dga1 fusion. *Asterisks* (∗) denote significantly different *LacZ* activity from NubG-Dga1 by Student’s *t* test, (∗*p* < 0.05, ∗∗*p* < 0.01, n.s. = not significant). Error bars reflect standard deviation. *C*, AlphaFold predicted structure of Dga1, and colors represent confidence of the predicted model. Residues in the carboxyl-terminal helix mutated from charged-to-alanine are indicated with *arrows*. *D*, HeliQuest plot of Dga1 carboxy-terminal helix, from Asp^382^ to Lys^406^. Negatively charged residues shown in *red*, positively charged residues shown in *blue*, polar residues shown in *pink* and *light blue*, and nonpolar residues shown in *yellow*.

The carboxyl terminus of Dga1 is essential for the catalytic activity of the enzyme, and deletion of residues 413 to 418 results in a near complete loss of activity despite Dga1 being retained in the ER (40). Dga1 lacks a traditional ER retention or retrieval signal at its carboxyl terminus, while it is possible that the small truncations at the carboxyl terminus inhibit localization this is unlikely as deletion of residues 413 to 418 do not reduce its retention in membranes (40). To determine whether the conserved DAELKIVG or KIVG motifs at the carboxyl terminus were important for maintaining the interaction between Dga1 and Ole1, in-frame deletions of residues 389 to 410, 399 to 410, and 399 to 414 were generated (Fig. 5*A*) Each version of Dga1 with these small in-frame internal deletions was assayed for interaction with Ole1. In each case, expression of the mutant version of *DGA1* allowed weak but detectable growth on the selective medium plates (Fig. 5*B*). The Δ389 to 410, Δ399 to 410, and Δ399 to 414 Dga1 deletions activated the LacZ reporter gene to a significantly lesser degree than full-length Dga1-inducing β-galactosidase activity to less than 20% of that induced by full-length NubG-Dga1 fusion (Fig. 5*B*). Indeed, the β-galactosidase activity driven by the internal deletion variants is similar to the larger Dga1_1–398_ truncation (Fig. 4*B*). These observations implied that mutations in Dga1 residues from 398 to 411 are sufficient to disrupt the interaction of Dga1 with Ole1.

The carboxy-terminal 20 amino acids of Dga1 feature several short segments that include charged residues. To determine if these charged segments might be important for the interaction of Dga1 with Ole1, we performed charged-to-alanine scanning mutagenesis. The residues chosen for this alanine scanning experiment are shown on the AlphaFold generated model where it can be seen that they cluster on a surface predicted to be exposed to the cytoplasm (Fig. 5*C*). The helix in the model contains Asp^382^ to Lys^406^ and is amphipathic in nature (Fig. 5, *C* and *D*). The predicted surface of this segment of Dga1 contains a series of charged residues, including Lys^397^, Arg^398^, Arg^404^, Glu^405^, and Lys^406^. The more hydrophobic face of this helix is oriented toward the predicted hydrophobic core of Dga1 and the highly conserved motif ^288^R*X*GF*X*(K/R)*X*A*XXX*G*XXX*(L/V)VP*XXX*FG(E/Q)^311^ (Fig. 5*C*) which is essential for DGAT activity (36, 40). When tested for interaction with Ole1 in the membrane two-hybrid system, Dga1 R^404^A, E^405^A, K^406^A (NubG-Dga1_REK_) displayed growth on selective medium and β-galactosidase activity that was not significantly different from wildtype Dga1 (Fig. 5*B*). In this assay, both of the mutants Dga1 K^397^A, R^398^A (NubG-Dga1_KR_) and Dga1 D^411^A, E^413^A, K^415^A (NubG-Dga1_DEK_) displayed reduced growth on selective medium relative to wildtype Dga1 (Fig. 5*B*). Similarly, both mutants displayed a significant reduction in β-galactosidase activity to about 60% of that induced by wildtype Dga1 (*p* = 0.006, *p* = 0.012 respectively) (Fig. 5*B*). Combining these mutations in NubG-Dga1 K^397^A, R^398^A, D^411^A, E^413^A, K^415^A (NubG-Dga1_KRDEK_) resulted in a discernable reduction in growth on selective medium and a significant decrease in β-galactosidase activity to a level similar to that displayed by deleting the last 20 amino acids of Dga1 (*p* = 0.004) (Fig. 5*B*). These data are consistent with the contention that the charged clusters in the carboxy-terminal 20 amino acids residues of Dga1 play a role in allowing the interaction of Dga1 and Ole1 *in vivo*.

### Interaction of Dga1 with Slc1 displays sequence requirements distinct from those required for Ole1

**Figure 6:**
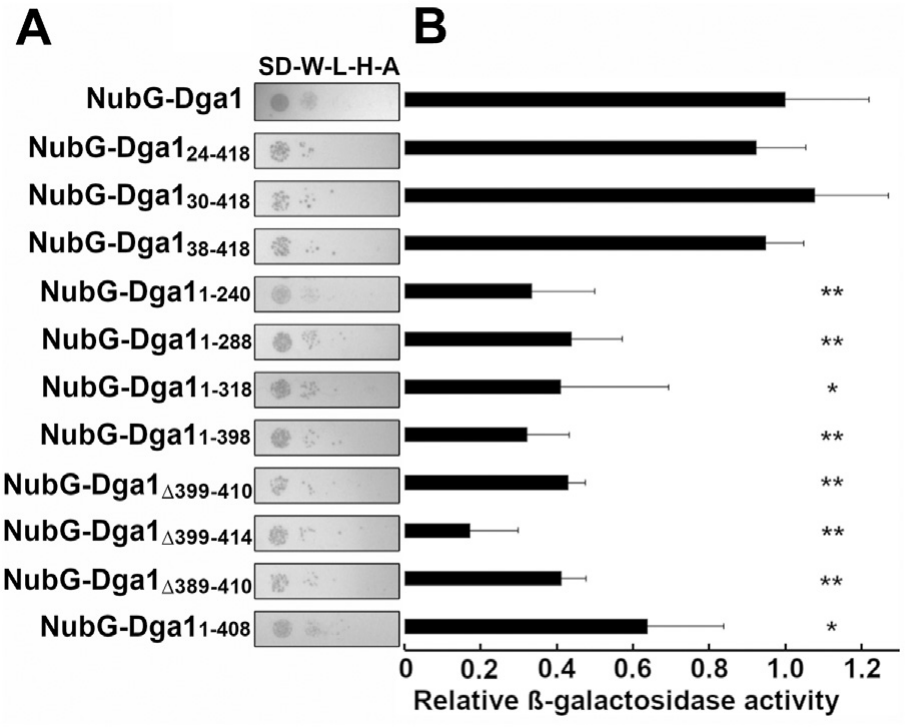
**Slc1 interacts with a truncated Dga1.***A*, membrane yeast two-hybrid assay using a yeast strain expressing Slc1-C_ub_-LexA-VP16 as “bait” and the indicated NubG-Dga1 fusions as “prey”. Growth on selective medium is representative of three independent experiments. Synthetic-defined medium lacking tryptophan, leucine, histidine, and adenine (SD-W-L-H-A) was used for the growth test. *B*, β-galactosidase activity in Miller units assayed from whole cell extracts assay of NMY51 expressing Slc1-C_ub_-LexA-VP16 and the indicated NubG-Dga1 fusions. Each column is representative of three biological replicates (n = 3). Miller units were normalized to the full-length NubG-Dga1 fusion. *Asterisks* (∗) denote significantly different *LacZ* activity from NubG-Dga1 by Student’s *t* test, (∗*p* < 0.05, ∗∗*p* < 0.01). Error bars reflect standard deviation.

Dga1 displays interactions with both the Δ9 desaturase Ole1 and acyltransferase Slc1. We were interested in investigating whether Dga1 bound to Slc1 through a similar sequence domain to that required for binding to Ole1. This was tested by performing a membrane two-hybrid test using the *SLC1*-Cub-LexA-VP16 fusion as “bait” for the NubG-Dga1 variants. Similar to the result observed with Ole1 as bait, the amino-terminal Dga1 truncations up to amino acid 37 displayed effective interaction with Slc1 based upon the reporter strains growth on selective medium and β-galactosidase activity similar to the full-length Dga1 (Fig. 6, *A* and *B*). In contrast, truncations of the carboxyl-terminal residues resulted in a reduction in the growth of the reporter strain on selective medium (Fig. 6*A*). These strains all displayed a reduction of β-galactosidase activity to levels 20 to 40% of that measured in reporter strains expressing the full-length NubG-Dga1 fusion (Fig. 6*B*). The reduction in binding to Slc1 displayed by the carboxyl-terminal truncations of Dga1 displays a similar pattern to that observed when binding to Ole1 was tested, but expression of the β-galactosidase reporter gene was reduced to a greater extent in the Ole1 binding test than observed in the Slc1 binding test.

### Dga1 charged-to-alanine mutants are defective in Ole1 binding but retain acyltransferase function

**Figure 7:**
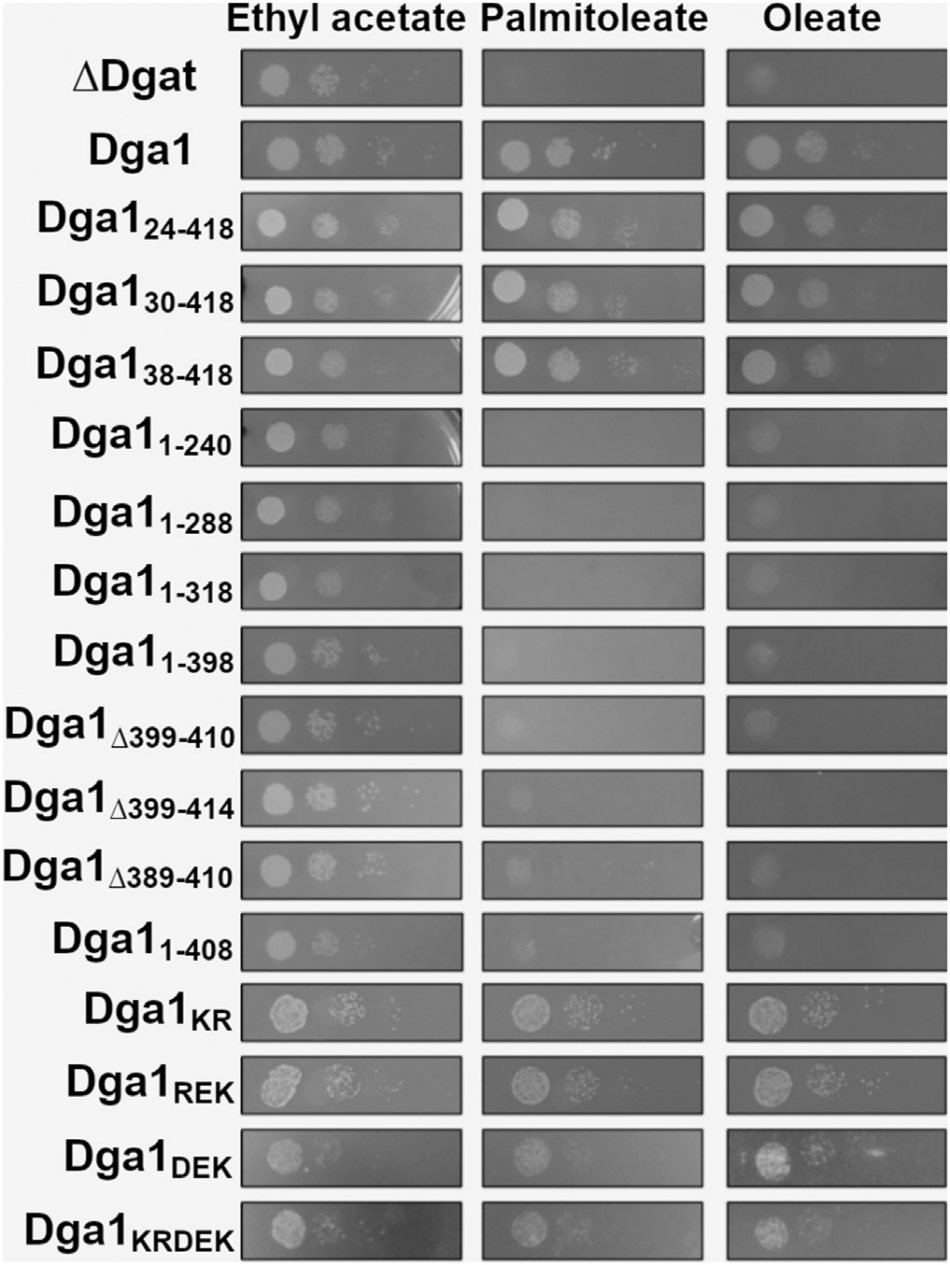
**Dga1 charged-to alanine variants are functional.** Fatty acid toxicity assay using medium supplemented with ethyl acetate (0.05%), palmitoleic acid (0.5 mM), or oleic acid (0.5 mM). The strain H1246 (*are1 are2 lro1 dga1*) harbored an empty vector (ΔDgat) or the indicated variants of *DGA1*. Strains were allowed to grow for 2 days at 30 °C before imaging.

Dga1 activity is not essential, and triglyceride synthesis is not a required activity for *S. cerevisiae* (43). However, acyltransferase activity does become important when cells are challenged with unsaturated free fatty acids (44). In the absence of acyltransferases Are1, Are2, Dga1, and Lro1, fatty acids become toxic to *S. cerevisiae* as displayed by the inability of H1246 cells that lack Are1, Are2, Dga1, and Lro1 to grow in the presence of 0.05 mM oleic or palmitoleic acid (Fig. 7, ΔDgat). Growth on this medium is restored by expression of full-length Dga1 presumably owing to its ability to sequester the unsaturated fatty acids into TAG (Fig. 7, Dga1). The truncated versions of Dga1 were tested to determine whether they had sufficient activity to rescue growth of the H1246 strain on medium supplemented with unsaturated fatty acids. All of the amino-terminal truncations could rescue growth in the presence of either oleic acid or palmitoleic acid, suggesting that they were active (Fig. 7). This observation supports the previous finding suggesting these truncated versions were properly localized to the ER membrane (Fig. 4*C*). In contrast, deletion of any amount of the carboxyl terminus of Dga1 eliminated fatty acid tolerance, suggesting that these variants of Dga1 are nonfunctional (Fig. 7). The correlation between Dga1 function and ability to interact with Ole1 was further tested by challenging H1246 strains expressing the Dga1 charged-to-alanine mutants with fatty acid–supplemented medium. Interestingly, H1246 cells expressing the alanine scanning mutants, including Dga1_KRDEK_ that was defective in Ole1 binding, were all able to grow on fatty acid supplemented medium similar to cells expressing full-length Dga1, indicating that these Dga1 variants are functional and able to incorporate fatty acids into TAG (Fig. 7).

**Figure 8:**
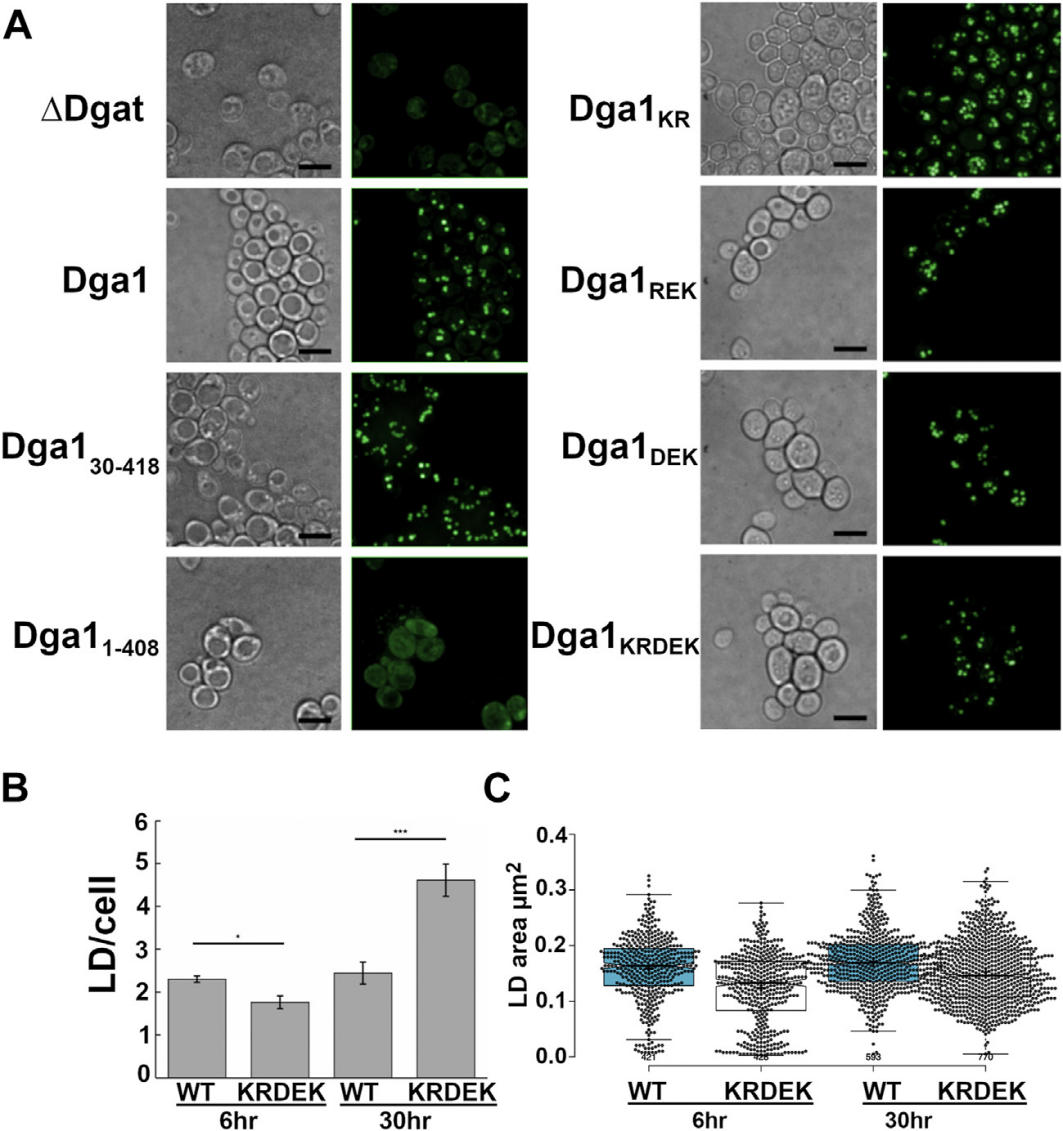
**Lipid droplet formation is altered by carboxyl-terminal mutations in Dga1.***A*, cultures of H1246 harboring the indicated HA-Dga1 variants were grown to stationary-phase, stained with BODIPY 493/503 and visualized by microscopic examination under white light (DIC) or epifluorescence. The micrometer marker bar in the DIC images represents 10 μm. Fluorescence images are Z projections of whole cells. *B*, lipid droplets visualized by microscopy were quantified in H1246 strains expressing *DGA1* (WT) or the charged-to-alanine mutant *DGA1*_KRDEK_ (KRDEK) at the indicated times. *Bars* indicate the mean from counting LDs detected in cells of each strain at each time point WT 6 h n = 166, KRDEK 6 h n = 188, WT 30 h n = 224, KRDEK 30 h n = 167. Error bars reflect standard deviation. *Asterisks* (∗) denote significantly different LD number from WT by Student’s *t* test, (∗*p* < 0.05, ∗∗*p* < 0.01, ∗∗∗*p* < 0.001). *C*, LD size was quantitated with the ALDQ Fili plug in method and data reflect mean size μm^2^ displayed as box and whisker plots for cells counted at each time point. WT 6 h n = 421, 30 h n = 593. KRDEK 6 h n = 426, 30 h n = 770. LD, lipid droplet.

The primary function of Dga1 is TAG synthesis leading to LD formation in *S. cerevisiae*. Microscopic analysis of *are1 are2 lro1 DGA1*_30–418_ strains stained with BODIPY demonstrated that LDs could be readily visualized (Fig. 8*A*). There did not appear to be any difference in the size or number of LDs per cell detected in *DGA1*_30–418_ when compared to full-length *DGA1* (Fig. 8*A*). In contrast, staining H1246 expressing *DGA1*_1–408_ with BODIPY did not reveal any LD formation, only diffuse staining could be detected (Fig. 8*A*). LDs could be detected in H1246 strains expressing the Dga1_KR_, Dga1_DEK,_ Dga1_REK,_ or Dga1_KRDEK_ charged to alanine variants consistent with those variants being functional (Fig. 8*A*). This indicates that the charged-to-alanine mutations do not disrupt Dga1 catalytic activity but rather have a specific effect on the ability to bind Ole1. These observations also indicate that the ability of Dga1 to bind Ole1 is not essential for Dga1 function and LD formation when unsaturated fatty acids are exogenously supplied. However, further analysis of BODIPY-stained cells revealed that Dga1_KRDEK_ expressing cells accumulated fewer LDs during early exponential growth than did cells expressing full-length Dga1 (Fig. 8*B*). In contrast, Dga1_KRDEK_ expressing cells accumulated more LDs when the cells approached early stationary phase (Fig. 8*B*). Quantification of LD size from confocal microscopy images revealed that although Dga1_KRDEK_ strains accumulated more LDs per cell, those LDs were smaller during both exponential growth (0.123 ± 0.064 μm^2^ *versus* 0.158 ± 0.059 μm^2^) and in early stationary phase (0.152 ± 0.060 μm^2^ *versus* 0.171 ± 0.053 μm^2^) than those in cells expressing Dga1 (Fig. 8*C*). To determine whether the TAG accumulation was affected by the charged to alanine mutations introduced into Dga1, we compared the amount of TAG in *are1 are2 lro1 DGA1* and *are1 are2 lro1 DGA1*_*KRDEK*_ strains that had reached early stationary phase growth. Quantification of TAG species separated by thin layer chromatography indicated that there was a reduction of 8.7 ± 3.4% in the TAG staining in the *DGA1*_*KRDEK*_ strain relative to the *DGA1* expressing strain (Fig. S2). Additionally, analysis of FAMEs from the two strains indicated that the C16/C18 ratio, 1.16 for Dga1 *versus* 1.13 for Dga1_KRDEK_, was unaffected by the mutations, but a decrease in the ratio of unsaturated to saturated acyl-chains was detected, 5.51 ± 0.01 *versus* 4.97 ± 0.18 for *DGA1* and *DGA*_*KRDEK*_ respectively.

These observations are consistent with the notion that compromising the interaction between Dga1 and Ole1 does not prevent the synthesis of TAG but may be disruptive to some aspects of LD formation and expansion.

## Discussion

Through the application of targeted membrane yeast two-hybrid (MYTH) assays, this investigation has revealed previously unrecognized interactions between the Δ9 acyl-CoA desaturase Ole1 and acyltransferases Sct1, Gpt2, Slc1, and Dga1. The interaction of these enzymes that all have roles in the synthesis of phospholipid and TAG suggests the possibility of a metabolon or enzyme supercomplex organization that could provide the benefits of substrate channeling to increase pathway flux in glycerophospholipid biosynthesis as well as facilitate control over intermediates that could disrupt membrane function should they accumulate inappropriately. Cross-linking experiments with human cell cultures have provided support for the contention that acyltransferases DGAT2 and monoacylglycerol transferase-2 reside in a high molecular weight complex that likely includes SCD1, but the organization of this complex is yet to be investigated (25). A benefit of the MYTH assay applied in this study is that a positive signal demands close physical interaction of the tested proteins and co-complex formation is not sufficient (45). Positive interactions were also detected between acyltransferases Gpt2–Slc1 and Slc1–Dga1 consistent with the presence of an acyltransferase interactome among enzymes involved in the synthesis of phospholipids and TAG in *S. cerevisiae*.

Physical interaction between Dga1 and Ole1 has not been previously reported in *S. cerevisiae*. There is however precedent for this interaction as the orthologs of *DGA1* and *OLE1* in human cells and *C. elegans* (DGAT2, SCD1) have been demonstrated to colocalize and human DGAT2 and SCD1 can be co-immunoprecipitated (32). Dga1 shuttles between the ER and LD dependent upon the growth stage of the cells (46, 47). This likely creates at least two distinct pools of Dga1 since Ole1 is present only in the ER. Similarly, human cells display two separate pools of DGAT2, one localized in the ER and one associated with LDs (36). The role of Dga1 at LDs is clearly to promote TAG synthesis and storage in the LD. It is less clear what function Dga1 serves while interacting with Ole1 in the ER membrane. Investigations in *C. elegans* indicate that retaining DGAT2 in the ER allows for LD initiation but fails to support LD expansion (48). This may imply that DGAT activity in the ER is necessary to initiate LD formation, but further expansion of the LD requires that DGAT activity be able to segregate from the ER to the LD membrane structures. It is notable that the second *S. cerevisiae* DGAT, Lro1, can support initiation of LD biogenesis but does not segregate to LDs to support their expansion leading to reduced LD formation in stationary phase strains lacking Dga1 (49).

Δ9 desaturase Ole1 has a central role in the synthesis of phospholipids and TAG. Acyl chains are synthesized *de novo* as saturated chains by the FAS complex, and formation of monounsaturated chains is dependent on Ole1 activity. Thus, a significant portion of newly synthesized acyl-CoA is destined to become substrate for Ole1. LD initiation is dependent upon incorporation of unsaturated acyl-chains into DAG and TAG (50). Thus, linking Dga1 to Ole1 may be a means to efficiently influence LD initiation as this places Dga1 in close proximity to both the source of *de novo* synthesized monounsaturated acyl chains and the sites of LD formation. This also positions Dga1 to rapidly move onto the monolayer membrane of growing LDs. We also find that the lysophosphatidic acid acyltransferase Slc1 interacts with Ole1 which could potentially create complexes including Slc1-Ole1-Dga1 that would be able to supply PA and DAG as well as unsaturated acyl-CoA for TAG synthesis. Dga1 and Slc1 can also interact with one another independent of Ole1 consistent with the presence of these two enzymes in the LD surface where they can promote TAG accumulation in the LD.

Interaction of Ole1 with Slc1 may be a means by which unsaturated acyl chains can be directed toward phospholipid synthesis in rapidly proliferating cells. The degree of unsaturation in membrane phospholipids is a critical determinant of membrane properties including membrane fluidity and domain structures that influence functionality of membrane embedded proteins. Interaction of Slc1 with Ole1 may also form a complex to receive LPA generated by glycerophosphate acyltransferases Sct1 and Gpt2. A physical interaction between Ole1 and overexpressed Gpt2 has previously been demonstrated by co-immunoprecipitation (51). Our observation that Sct1 and Gpt2 display interaction with Ole1 provides further support for this contention. Placing Sct1, Gpt2, and Slc1 in close proximity may aid in channeling acyl-CoA to synthesize PA species that can be further processed to yield the major membrane phospholipids.

Mutational analysis of Dga1 revealed that the carboxyl terminus, predicted to form a cytoplasmic domain, is required for association with Ole1 but not for membrane localization. Truncations and in-frame internal deletions support a model indicating that interaction with Ole1 is mediated by sequences between Leu^396^ to Gly^408^. The final ten amino acids residues 408 to 418 of Dga1 are required for catalytic activity and their deletion reduced but did not eliminate binding to Ole1. The segment from L^396^ to K^415^ encodes a several of clusters of charged residues. Mutation of Lys^397^, Arg^398^, Asp^411^, Glu^413^, and Lys^415^ to alanine leads to a near complete ablation of interaction with Ole1. Although we cannot state with confidence that Dga1_KRDEK_ has wildtype levels of enzymatic activity, it does retain catalytic activity and function based on the ability to rescue fatty acid–induced toxicity and promote formation of LDs, thus the loss of interaction with Ole1 is not due to global misfolding or mislocalization.

Although there is no experimentally derived structure for Dga1, the proposal that the carboxyl-terminal domain of Dga1 provides a surface for interaction with Ole1 is consistent with the AlphaFold model that places the charged residues Lys^397^, Arg^398^, Asp^411^, Glu^413^, and Lys^415^ on an alpha-helix structure arranged on the surface oriented away from the core of Dga1 in a position suitable to make contacts with other proteins. Our data provide no mechanistic information for how the carboxyl terminus of Dga1 interacts with Ole1. While the charge of the helix surface may be responsible for the interaction, we cannot discount a specific structure formed by this domain being responsible or even the possibility that posttranslational modification of carboxyl-terminal residues is responsible. Modification to the carboxyl-terminal amino acids of Dga1 has not been reported, but Dga1 is subject to regulation by the ER-associated degradation system (52). The sequences that target Dga1 for destabilization have not been identified, but lysine residues in the carboxyl terminus of mammalian DGAT2 are ubiquitinated to trigger DGAT2 destruction and mutation of those residues to alanine stabilize DGAT2 (53).

The *DGA1*_*KRDEK*_ mutant has reduced ability to interact with Ole1 but is active in the production of TAG and can channel exogenous fatty acids into TAG stored in LDs, thus preventing lipotoxicity in response to exogenous unsaturated fatty acids. In comparison with *are1 are2 lro1 DGA1* strain, the *are1 are2 lro1 DGA1*_*KREDK*_ cells produce fewer detectable LDs, and they are smaller during early exponential growth. Untethering Dga1 from Ole1 may disrupt or destabilize the localization of Dga1 to sites of LD initiation, and the channeling of endogenously produced unsaturated acyl-CoA to DAG leading to delayed initiation and growth of LDs.

In contrast, as cells approach quiescence, the *DGA1*_*KRDEK*_ strain displays more LDs than the *DGA1* strain but they are smaller. This is a puzzling observation and may reflect our limited understanding of LD biogenesis. One possibility is that this reflects reduced efficiency in channeling unsaturated acyl-CoA to sites of LD growth but since unsaturated acyl-CoA continues to accumulate, ER membrane–associated Dga1_KRDEK_ would continue to participate in TAG production, leading to the formation of more LDs. A more trivial explanation that we cannot categorically eliminate is that a minor reduction in Dga1 activity caused by the charged-to-alanine mutations combined with the loss of Ole1 binding may be responsible for the alteration in LD initiation and growth. In the absence of further experimental data, the mechanism responsible for this observation remains conjecture since the alterations to Dga1 may influence other as yet undetected functions or protein interactions that may lead to disruption of LD initiation or biogenesis.

The interactions we detect between Ole1 and acyltransferases may also be a reflection of the importance of directing lipid species to appropriate fates and the potential for toxicity caused by ER accumulation of DAG (49). Even though the loss of all DGAT activities is not lethal to *S. cerevisiae*, it does result in alteration in membrane structure and function owing to the accumulation of DAG in the ER (49). Placing Dga1 in close proximity to Ole1 may be a means to capture any overflow DAG and unsaturated acyl-CoA to avoid accumulation in the ER membrane. Indeed, increased Ole1 activity yielding increased unsaturated acyl-CoA or a reduction in Cds1 activity yielding decreased flux of unsaturated acyl-CoA to phospholipids leads to an increase in LD formation (54). Cells with reduced capacity for TAG synthesis and LD formation are very sensitive to increased Ole1 or reduced Cds1 activity (54). Thus, reversibly tethering Dga1 to Ole1 in the ER may create a pathway to allow rapid response to excess accumulation of DAG and unsaturated acyl-CoA, channeling these into neutral lipid storage in LDs.

## Experimental procedures

### Strains and plasmids

The *S. cerevisiae* strain NMY51 (*MATa, his3Δ200, trp1-901, leu2- 3,112, ade2, LYS2::(lexAop)4-HIS3,ura3::(lexAop)8-LacZ, ade2::(lexAop)8-ADE2, GAL4*) was provided by Marek Michalak for the MYTH experiments. Oligonucleotides and synthetic DNA fragments used in this investigation are listed in Table S1. Plasmids used in this study are listed in Table S2. To introduce the Cub-LexA-VP16 tag into the endogenous *OLE1* gene in NMY51 (YBG1), sequence encoding the *Cub-LexA-VP16* tag containing the *CYC1* terminator was amplified from plasmid pTMBV4 (Dualsystems Biotech AG) using primers 5-Cub and 3-Cub. The 5′ oligonucleotide (5-Cub) included sequence to insert a single Myc epitope and SpeI cleavage site immediately upstream of the Cub sequence. The resulting DNA fragment was digested with SpeI and phosphorylated using T4 polynucleotide kinase prior to ligation with SpeI – EcoRV digested pUG6 (55) resulting in pUG6-CLVt. The *MYC-Cub-LexA-VP16-CYC1t-KanMX* fragment was amplified from pUG6-CLVt with primers (OLE1t-5, OLE1t-3) containing homology to *OLE1*. This DNA fragment was used to transform NMY51. Transformants were selected on YEPD agar supplemented with 200 μg/ml geneticin, and correct integration of Ole1-Myc-Cub-LexA-VP16 was confirmed by PCR analysis of genomic DNA using oligonucleotides BGO15 and BGCub3-1. YBG3 was derived from NMY51 by deletion of the endogenous *OLE1* gene. The Nat-MX6 gene was amplified from pAG25 (55) using primers Ole1d5 and Ole1d3 the amplified DNA fragment containing homology to *OLE1* was used to transform NMY51, and transformants were selected on YEPD agar supplemented with 100 μg/ml nourseothricin and 0.5% Tween-80. The lack of *OLE1* function was confirmed by PCR analysis of genomic DNA using primers Ole1dsc5 and Ole1dsc3 and lack of growth on YEPD plates supplemented with 100 μg/ml nourseothricin but lacking Tween-80.

To produce the *SLC1*-Cub-LexA-VP16 fusion, the *SLC1* coding sequence was amplified from W303 genomic DNA using primers pTMBV4-SLC1f and pTMBV4-SLC1r and inserted into pTMBV4 plasmid in frame with the Cub-LexA-VP16 tag using Gibson isothermal assembly (56). Correct assembly of pTMBV4-SLC1 was confirmed by sequencing. The NubG fusions used for two-hybrid testing were constructed using plasmids pADSL-Nx and pADSL-xN digested with BamHI and EcoRI. *DGA1* and *DGA1* variants amplified with truncations of the 5′- and 3′-sequences from W303 genomic DNA were inserted in frame with *NubG-HA*. For production of the pADSL-NubG-*SLC1* plasmid, the *SLC1* open reading frame was amplified from *S. cerevisiae* genomic DNA using primers SLC1f and SLC1r and inserted in frame with *NubG-HA*. For production of the pADSL-NubG-*SCT1* and pADSL-NubG-*GPT2* vectors, *SCT1* and *GPT2* coding sequences were amplified from *S. cerevisiae* genomic DNA using primer pairs SCT1f - SCT1r and GPT2f - GPT2r. The resulting DNA fragments were inserted in frame with *NubG-HA*. To create the pADSL-SCT1-NubG construct, *SCT1* was amplified from *S. cerevisiae* genomic DNA using primers SCT1xNf and SCT1xNr. and ligated in to a BamHI – SfiI digested pADSL-xN in frame with *HA-NubG*.

*DGA1*_*S17A*_ and *DGA1*_*S17D*_ were generated by splice overlap PCR using oligonucleotides. S17Af/S17Ar and S17Df/S17Dr. Dga1 carboxyl-terminal truncations were generated by PCR amplification of *DGA1* coding sequence using oligonucleotides 212, 240, 288, 318, 388, and 398. Similarly amino-terminal truncations were generated by amplification of *DGA1* with primers DGA1-23f, DGA1-29f, and DGA1-37f. In-frame internal deletions Δ399 to 410, Δ399 to 414, and Δ389 to 410 were generated using oligonucleotides DGAD398, DGAD398a, and DGAD388. Charged-to-alanine mutations in *DGA1* were generated using synthetic DNA fragments (Table S1). Each DNA fragment was assembled with StuI/EcoRI digested pADSL-NubG-DGA1. All DNA constructs were confirmed by DNA sequencing.

The W303-derived H1246 (*MAT*α *are1*-Δ::*HIS3 are2*-Δ::*LEU2 dga1*-Δ::*KanMX4 Iro1*-Δ::*TRP1 ADE2 ura3*) (43) was obtained from Dr Randall Weselake for the fatty acid toxicity analysis. The *DGA1* variants used for functional testing were placed under the regulation of the native *DGA1* promoter in integrating vector YIplac211. The promoter was amplified from W303 genomic DNA with a forward primer containing homology to the Pst1 cut site on YIplac211 (YI-DGA1pf, DGA1pr), and the HA-*DGA1* variants were amplified from the respective pADSL-*DGA1* plasmids using a forward primer for *DGA1* with homology to the *DGA1* promoter and a reverse primer for *CYC1t* with homology to the PstI cut site on YIplac211 (DGA1p-HAf, YI-PSTCYC1). The promoter and *DGA1* truncations were inserted into the vector by Gibson isothermal assembly to produce the YIplac211-*DGA1* variants. These were digested with EcoRV to direct integration at the *URA3* locus and used to transform H1246. All yeast transformations were completed with the lithium acetate method (57). *Escherichia coli* DH5α was used in all cloning steps and for routine propagation of all plasmids.

### Medium and cultivation conditions

*E. coli* strains were cultivated in lysogeny broth containing ampicillin (100 μg/ml) or kanamycin (50 μg/ml) as needed for plasmid maintenance. Yeast strains were propagated on YEPD medium (1% yeast extract, 2% peptone, 2% dextrose) or on synthetic defined medium (0.17% yeast nitrogen base without amino acids without ammonium sulfate, 0.5% ammonium sulfate, 2% dextrose, supplemented with an amino acid mixture lacking the amino acids or purines as required for selection).

### Membrane yeast two-hybrid assay

Interaction between specific proteins was tested using the MYTH test essentially as described (45). The reporter strains NMY51 and YBG1 (OLE1-Cub-LexA-VP16) were validated by transformation with positive and negative control vectors expressing Alg5-NubI and Alg5-NubG. The concentration of 3-aminotriazole necessary to remove background strain growth on medium lacking histidine was determined to be 6 mM. YBG1 harboring the integrated *OLE* bait was transformed with the prey plasmids and selected on synthetic minimal agar plates lacking tryptophan (-trp) to ensure retention of the vectors. Plasmid-borne bait and prey pairs were tested in NMY51 by transformation of both plasmids and selection on medium lacking both tryptophan and leucine (-trp -leu). In both cases, interaction was assayed on synthetic minimal agar plates lacking tryptophan and histidine and adenine, supplemented with 6 mM 3-aminotriazole. Spot assays were performed by spotting cultures serially diluted 1:10 starting from 1.0 × 10^4^ cells.

*LacZ* expression was assayed by β-galactosidase assay. Strains were cultured in -trp or -trp -leu liquid media overnight at 30 °C with agitation. The culture density was determined based on absorbance at 600 nm (*A*_*600*_), and 1 ml of each culture was harvested by centrifugation. The cell pellets were resuspended in 500 μl Z-buffer (60 mM Na_2_HPO_4_, 40 mM NaH_2_PO_4_, 10 mM KCl, 1 mM MgSO_4_) and 50 μl 0.1% SDS by vortexing. Chloroform (100 μl) was added, and the mixture was vortexed for 15 s before adding 100 μl of 4 mg/ml ortho-nitrophenyl-β-galactoside. The reaction mixture was incubated at 37 °C until color development and then quenched by addition of 500 μl 1 M Na_2_CO_3_. Reactions were centrifuged to remove cell debris, and the color development was assayed by measuring *A*_*420*_. β-galactosidase activity is presented as the mean of three independent colonies normalized either to the positive control, Alg5-NubI, or to the full-length NubG-Dga1 as indicated. Significance was determined using a two-tailed *t* test assuming equal variance.

### Protein extraction and Western blotting

Expression of all bait and prey proteins was confirmed by Western blot. Proteins were extracted by bead beating in trichloroacetic acid as described (58), and equivalent volumes of protein were resolved using 10% SDS-PAGE. Proteins were electrotransferred to polyvinylidene difluoride membranes. The prey constructs were detected using anti-HA monoclonal antibody HA.11 clone 16B12 mouse ascites fluid and goat anti-mouse conjugated to horseradish peroxidase (HRP) secondary antibody. Cdk1 was used to determine relative amounts of protein loaded and was detected with an anti-PSTAIR antibody (P7962 Sigma-Aldrich) and goat anti-mouse HRP secondary antibody. The relative band densities of the NubG-Dga1 variants were quantified using BioRad Image Lab analysis software. The Ole1-Cub-LexA-VP16 fusion was detected with a rabbit polyclonal anti-LexA primary antibody (06-719 EMD Millipore) and goat anti-rabbit HRP secondary antibody.

### Fatty acid toxicity assay

Fatty acid tolerance studies were performed both on agar-containing plates and in liquid medium. Synthetic minimal agar plates and liquid media lacking uracil (-ura) and containing 0.05% ethyl acetate and 0.5 mM of the indicated fatty acid were used. For the plate-based fatty acid toxicity assays, cultures were inoculated from a single colony and allowed to grow overnight in -ura media. Cultures were spotted to the selective medium plates starting from 1.0 × 10^4^ cells, diluted 1:10 serially. Images were obtained following 48 h of incubation at 30 °C. Fatty acid toxicity was assayed in liquid culture by inoculating cultures into SD medium lacking uracil from a single colony and growing overnight. Cultures were normalized to *A*_*600*_ 0.05 in 1 ml of the indicated media and assayed in triplicate using biological replicates. Cells were cultured in a 2 ml deep well plate for 30 h, using a plate shaker shaking at 900 rpm at 30 °C. Samples were taken every 6 h and analyzed on a Spectra Max M3, using the respective media as a blank.

### Lipid analysis

TAG accumulation was assayed by TLC analysis of total cellular lipid species on silica gel plates as previously described (59). TAG species visualized on TLC plates were quantified using Image J. Total cellular lipid accumulation was further corroborated by whole cell staining with Nile red as previously described (59). FAME analysis was performed by gas chromatography with an Agilent 6890 GC instrument equipped with flame ionization detector as previously described (60).

### Confocal microscopy

Strains were cultured in YEPD medium at 30 °C overnight. Cells were diluted into fresh YEPD supplemented with 100 μg/ml adenine at an *A*_600_ of 0.2 and cultured for the indicated time periods. Cultures for LD visualization were stained for 10 min in the dark using BODIPY 493/503 at a concentration of 50 μg/ml. All samples were washed in PBS and immobilized on 2% agarose pads prior to imaging. Images were collected on a Yokagawa CSU-X1 microscope using the GFP laser and filter, a Hamamatsu EMCCD (C9100-13) camera, and PerkinElmer Volocity software. Images were analyzed using Fiji and LD number and size were determined with the ALDQ plugin (61). LD number and size were quantified from at least 100 cells.

### Statistical analysis

The data are presented as mean values, and error bars reflect standard deviation. All n values are indicated in the figure legends. Statistical significance was evaluated by paired, two-tailed *t* test. Differences in LD number were tested by one-way ANOVA performed with MATlab. Statistical significance is depicted in figures or noted in the figure legends (∗ represents *p* < 0.05, ∗∗*p* < 0.01, ∗∗∗*p* < 0.001). Box and whisker plots to display data were constructed using BoxplotR.

## Data availability

All of the data are contained within the manuscript and the supporting information.

## Supporting information

This article contains supporting information.

## Conflict of interest

The authors declare that they have no conflicts of interest with the contents of this article.
