# Supplementary material for: Saccharomyces cerevisiae Δ9-desaturase Ole1 forms a supercomplex with Slc1 and Dga1
Source: J Biol Chem. 2023 Jun 1;299(7):104882. doi: 10.1016/j.jbc.2023.104882 (PMC10302205; doi:10.1016/j.jbc.2023.104882)
Supplement: Supporting information [file mmc1.pdf]

Saccharomyces cerevisiae ∆9-desaturase Ole1 forms a supercomplex with acyltransferases Slc1 and Dga1

Brianna L. Greenwood, Zijun Luo, Tareq Ahmed, Daniel Huang, and David T. Stuart#

Supporting information:
Table S1 Oligonucleotides and synthetic DNA used in this study
Table S2. Plasmids used in this study.
Figure S1. Western blot confirmation of bait and prey expression.
Figure S2. Thin Layer Chromatography analysis of TAG species.

Table S1 Oligonucleotides and synthetic DNA used in this study

DGA15	TTACGCTGGATCCATGTCAGGAACATTCAATGATAT
DGA1Nx3	TACGCTGGATCCGAAAGGCATGAGAATAAGTCT
DGA1xN3	GCAGAATTGAAGATAGTTGGGGAATTCGATATC
DGA123f	TACGCTGGATCCGAAAGGCATGAGAATAAGTCT
DGA129f	TACGCTGGATCCTCTTTGTCAAGCATCGATAA
DGA137f	TACGCTGGATCCGAACAGACTCTCAAACCACA
212r	GATATCGAATTCTTAGTTACAACCTTCTGTTGCA
240r	GATATCGAATTCTTATAAGTAGTCTCTATACAATGGGATAT
288r	GATATCGAATTCTTATCTTTTGTTTAAAATCAGTTGTGTAC
318r	GATATCGAATTCTTACAGAACATTATAACAGTCCACC
388r	GACGGTATCGATAAGCTTGATATCGAATTCTTATCTTTTCAACTCCGCAATATAC
398r	GACGGTATCGATAAGCTTGATATCGAATTCTTACCCATATTTTTCTCTATTTTCG
DGAD398	ATATCGAATTCTTACCCAACTATCTTCAATTCTGCATCTCTTTTCAACTCCGCAATATACAAAT CATG
DGAD398a	ATATCGAATTCTTACCCAACTATCTTTCTTTTCAACTCCGCAATATACAAATCATG
DGAD388	ATATCGAATTCTTACCCAACTATCTTCAATTCTGCATCGAAATGATTAACAACATCATCTGG
S17Df	GACCCTACAGCCGGTATTACC
S17Af	GCCCCTACAGCCGGTATTACC
S17Dr	CGGTAATACCGGCTGTAGGGTCTCCTTCTTCCTT
S17Ar	TAATACCGGCTGTAGGGGCTCCTTCTTCCT
TMBV4SLCf	AGCATAGCAATCTAATCTAAGTTTCTAGATGAGTGTGATAGGTAGGTTCTT
TMBV4SLCr	CGAATTCCTGCAGATATACCCATGGAGATGCATCTTTTTTACAGATGAACCTT
SCT1f	TACGCTGGATCCCCTGCACCAAAACTCACG
SCT1r	CTACGCATCTCCTTCTTTCCC
SCT1xNf	TACGCTGGATCCATGCCTGCACCAAAACT
SCT1xNr	TACGCTGGCCgaggcgGCCGCATCTCCTTCTTTCC
SLC1f	TACGCTGGATCCAGTGTGATAGGTAGGTTCTTGTATTACT
SLC1r	GATATCGAATTCTTAATGCATCTTTTTTACAGATGAACCTTC
GPT2f	TACCCATACGATGTTCCAGATTACGCTGGATCCATGTCTGCTCCCGCTGCC
GPT2r	GACGGTATCGATAAGCTTGATATCGAATTCTCATTCTTTCTTTTCGTGTTCTCTTTTC TGTCTTACCAG
5-Cub	CGATAAACTAGTATGGAACAAAAACTTATTTCTGAAGAAGATCTGTCGACCATGTCGG
3-Cub	CGATAAGAATTCTAATACGACTCACTATAGGG
ole1T5	CTCTGCTATTAGAATGGCTAGTAAGAGAGGTGAAATCTACGAAACTGGTAAGTTCTTT GAACAAAAACTTATTTCTG
ole1T3	AGCATGACTGTGAAATTTTGAAGAGATGCAGTAAGCCATCCCATATCTATTGCTCCAG GGccagatctgtttagcttgc
ole1d3	TTATGGTAGTTGCAGTTTTGTTATTGTAATGTGATACTTAAAAGAACTTACCAGTTTC GTGCATAGGCCACTAGTGGATC
ole1d5	CATAGTAATAGATAGTTGTGGTGATCATATTATAAACAGCACTAAAACATTACAACAA AGCAGCTGAAGCTTCGTACGC
ole1dsc5	ATGCAGCAAATCATCGGCTC
ole1dsc3	AGAGATGCAGTAAGCCATCC
BGO15	TACGACTTGAAGAAATTCTCTC
BGCub3-1	CTTAGCACAAGATGTAAGGT
YI-DGA1pf	CAGGAAACAGCTATGACCATGATTACGCCAATCAACTAAGAGACCGTGGT

DGA1pr	TTATGTGACTGTTCAAACTGTATGC
DGA1p-HAf	CAGAGGCAGAGGCATACAGTTTGAACAGTCACATAAATGCCATACCCATACGATGTTC<br>C
YI-PstCYC1	TACCCGGGGATCCTCTAGAGTCGACCTGCAACCGGCCGCAAATTAAA

Synthetic DNA fragments

DGA1$_{KR}$	GATTTCGGTTTGTTGCCATTTAGAGCGCCTATCAATGTTGTTGTTGGAAGGCCTATATACGTTGA<br>AAAGAAAATAACAAATCCGCCAGATGATGTTGTTAATCATTTCCATGATTTGTATATTGCGGAGT<br>TGgcAgctCTATATTACGAAAATAGAGAAAAATATGGGGTACCGGATGCAGAATTGAAGATAGTT<br>GGGTAAGAATTCgatatcaagcttatcgataccgtcgacctcgagtcatgtaattag
DGA1$_{REK}$	GATTTCGGTTTGTTGCCATTTAGAGCGCCTATCAATGTTGTTGTTGGAAGGCCTATATACGTTGA<br>AAAGAAAATAACAAATCCGCCAGATGATGTTGTTAATCATTTCCATGATTTGTATATTGCGGAGT<br>TGAAAAGACTATATTACGAAAATgcaGctgccTATGGGGTACCGGATGCAGAATTGAAGATAGTT<br>GGGTAAGAATTCgatatcaagcttatcgataccgtcgacctcgagtcatgtaattag
DGA1$_{DEK}$	GATTTCGGTTTGTTGCCATTTAGAGCGCCTATCAATGTTGTTGTTGGAAGGCCTATATACGTTGA<br>AAAGAAAATAACAAATCCGCCAGATGATGTTGTTAATCATTTCCATGATTTGTATATTGCGGAGT<br>TGAAAAGACTATATTACGAAAATAGAGAAAAATATGGGGTACCGGcTGCAGcATTGgctATAGTT<br>GGGTAAGAATTCgatatcaagcttatcgataccgtcgacctcgagtcatgtaattag
DGA1$_{KRDEK}$	GATTTCGGTTTGTTGCCATTTAGAGCGCCTATCAATGTTGTTGTTGGAAGGCCTATATACGTTGA<br>AAAGAAAATAACAAATCCGCCAGATGATGTTGTTAATCATTTCCATGATTTGTATATTGCGGAGT<br>TGgcAgctCTATATTACGAAAATAGAGAAAAATATGGGGTACCGGcTGCAGcATTGgctATAGTT<br>GGGTAAGAATTCgatatcaagcttatcgataccgtcgacctcgagtcatgtaattag

Table S2. Plasmids used in this study

**Plasmid Name**	**S.c. Gene**	**Ori and selection**	**Source**
pTMBV4	*Cub-LexA-VP16*	*2μ LEU2*	Dual Systems
pADSL-Nx	*NubG*	*CEN/ARS TRP1*	Dual Systems
pADSL-xN	*NubG*	*CEN/ARS TRP1*	Dual Systems
pALG5-NubG	*NubG*	*CEN/ARS TRP1*	Dual Systems
pALG5-NubI	*NubI*	*CEN/ARS TRP1*	Dual Systems
pUG6	KanMX6	G418^{R}	Euroscarf
pUG6-CLVt	*Cub-LexA-VP16-KanMX6*	G418^{R}	This study
pAG25	*NatMX6*	ClonNAT^{R}	Euroscarf
pTMBV4-SLC1	*SLC1-Cub-LexA-VP16*	*2μ LEU2*	This study
DGA1-NubG	*DGA1-HA-NubG*	*CEN/ARS TRP1*	This study
NubG-DGA1	*NubG-HA-DGA1*	*CEN/ARS TRP1*	This study
NubG-SCT1	*NubG-HA-SCT1*	*CEN/ARS TRP1*	This study
SCT1-NubG	*SCT1-HA-NubG*	*CEN/ARS TRP1*	This study
NubG-GPT2	*NubG-HA-GPT2*	*CEN/ARS TRP1*	This study
NubG-SLC1	*NubG-HA-SLC1*	*CEN/ARS TRP1*	This study
NubG-DGA1 $_{24-418}$	*NubG-HA-DGA1* $_{24-418}$	*CEN/ARS TRP1*	This study
NubG-DGA1 $_{30-428}$	*NubG-HA-DGA1* $_{30-428}$	*CEN/ARS TRP1*	This study
NubG-DGA1 $_{38-418}$	*NubG-HA-DGA1* $_{38-418}$	*CEN/ARS TRP1*	This study
NubG-DGA1 $_{1-212}$	*NubG-HA -DGA1* $_{1-212}$	*CEN/ARS TRP1*	This study
NubG-DGA1 $_{1-240}$	*NubG-HA -DGA1* $_{1-240}$	*CEN/ARS TRP1*	This study
NubG-DGA1 $_{1-288}$	*NubG-HA -DGA1* $_{1-288}$	*CEN/ARS TRP1*	This study
NubG-DGA1 $_{1-318}$	*NubG-HA -DGA1* $_{1-318}$	*CEN/ARS TRP1*	This study
NubG-DGA1 $_{1-398}$	*NubG-HA -DGA1* $_{1-398}$	*CEN/ARS TRP1*	This study
NubG-DGA1 $_{\Delta 388-410}$	*NubG-HA-DGA1* $_{\Delta 388-410}$	*CEN/ARS TRP1*	This study
NubG-DGA1 $_{\Delta 399-410}$	*NubG-HA -DGA1* $_{\Delta 399-410}$	*CEN/ARS TRP1*	This study
NubG-DGA1$_{\Delta 399-414}$	*NubG-HA -DGA1*$_{\Delta 399-414}$	*CEN/ARS TRP1*	This study
NubG-DGA1 $_{1-408}$	*NubG-HA -DGA1* $_{1-408}$	*CEN/ARS TRP1*	This study
NubG-DGA1$_{KR}$	*NubG-HA -DGA1*$_{KR}$	*CEN/ARS TRP1*	This study
NubG-DGA1$_{REK}$	*NubG-HA -DGA1*$_{REK}$	*CEN/ARS TRP1*	This study
NubG-DGA1$_{DEK}$	*NubG-HA -DGA1*$_{DEK}$	*CEN/ARS TRP1*	This study
NubG-DGA1$_{KRDEK}$	*NubG-HA -DGA1*$_{KRDEK}$	*CEN/ARS TRP1*	This study
NubG-DGA1$_{S17A}$	*NubG-HA -DGA1*$_{S17A}$	*CEN/ARS TRP1*	This study
NubG-DGA1$_{S17D}$	*NubG-HA -DGA1*$_{S17D}$	*CEN/ARS TRP1*	This study
YIp-DGA1	*HA-DGA1*	*YIp URA3*	This study
YIp -DGA1 $_{24-418}$	*HA -DGA1* $_{24-418}$	*YIp URA3*	This study
YIp-DGA1 $_{30-428}$	*HA-DGA1* $_{30-428}$	*YIp URA3*	This study
YIp-DGA1 $_{38-418}$	*HA-DGA1* $_{38-418}$	*YIp URA3*	This study
YIp -DGA1 $_{1-212}$	*HA -DGA1* $_{1-212}$	*YIp URA3*	This study
YIp-DGA1 $_{1-240}$	*HA-DGA1* $_{1-240}$	*YIp URA3*	This study
YIp-DGA1 $_{1-288}$	*HA-DGA1* $_{1-288}$	*YIp URA3*	This study
YIp-DGA1 $_{1-318}$	*HA-DGA1* $_{1-318}$	*YIp URA3*	This study
YIp-DGA1 $_{1-398}$	*HA-DGA1* $_{1-398}$	*YIp URA3*	This study

YIp-DGA1 $_{\Delta 388\text{-}410}$	*HA-DGA1* $_{\Delta 388\text{-}410}$	*YIp URA3*	This study
YIp-DGA1 $_{\Delta 399\text{-}410}$	*HA-DGA1* $_{\Delta 399\text{-}410}$	*YIp URA3*	This study
YIp-DGA1$_{\Delta 399\text{-}414}$	*HA-DGA1*$_{\Delta 399\text{-}414}$	*YIp URA3*	This study
YIp-DGA1 $_{1\text{-}408}$	*HA- DGA1* $_{1\text{-}408}$	*YIp URA3*	This study
YIp-DGA1$_{KR}$	*HA-DGA1* $_{K397A,\ R398A}$	*YIp URA3*	This study
YIp-DGA1$_{REK}$	*HA-DGA1* $_{R404A,\ E405A,\ K406A}$	*YIp URA3*	This study
YIp-DGA1$_{DEK}$	*HA-DGA1* $_{D411A,\ E413A,\ K415A}$	*YIp URA3*	This study
YIp-DGA1$_{KRDEK}$	*HA-DGA1* $_{K397A,\ R398A,\ D411A,\ E413A,\ K415A}$	*YIp URA3*	This study
YIp-DGA1$_{S17A}$	*HA-DGA1* $_{S17A}$	*YIp URA3*	This study
YIp-DGA1$_{S17D}$	*HA-DGA1* $_{S17D}$	*YIp URA3*	This study

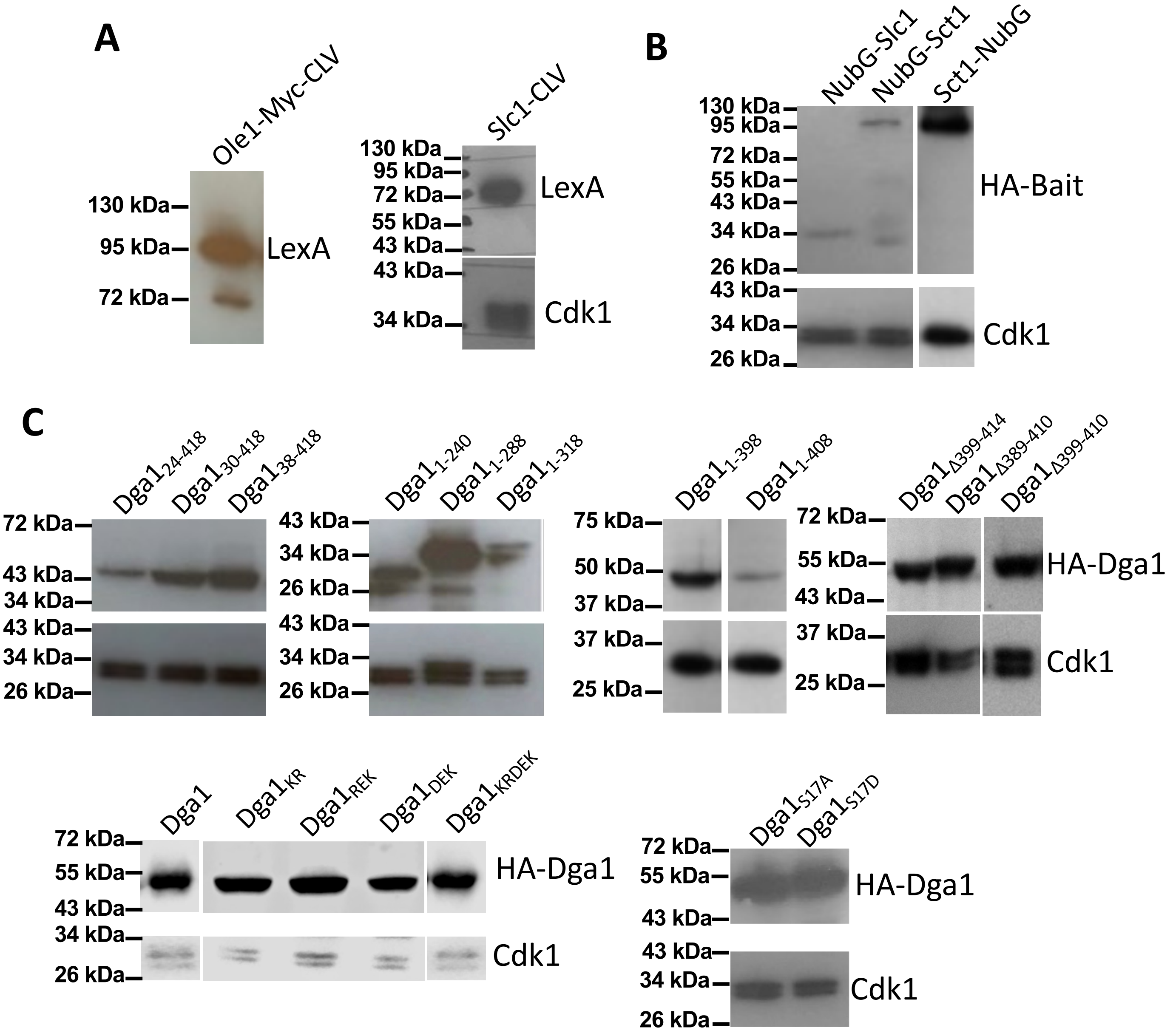

Figure S1. Western blot confirmation of bait and prey protein expression for two-hybrid testing. **A**. Ole1 and Slc1 fused to the carboxyl-terminus of ubiquitin-LexA-VP16 (CLV) were detected with an antibody against recognizing LexA. The blots were subsequently probed for Cdk1 with an antibody recognizing the PSTAIRE motif as a loading control. **B.** ”Prey” proteins Slc1 and Sct1 fused to NubG were visualized by probing blots with an antibody recognizing the HA epitope. **C**. Dga1 and Dga1 variants fused to NubG were all detected using an antibody recognizing the HA epitope. Cdk1 used as a loading control was detected using an anti-PSTAIRE antibody. The position to which pre-stained molecular weight markers migrated is indicated next to each set of lanes.

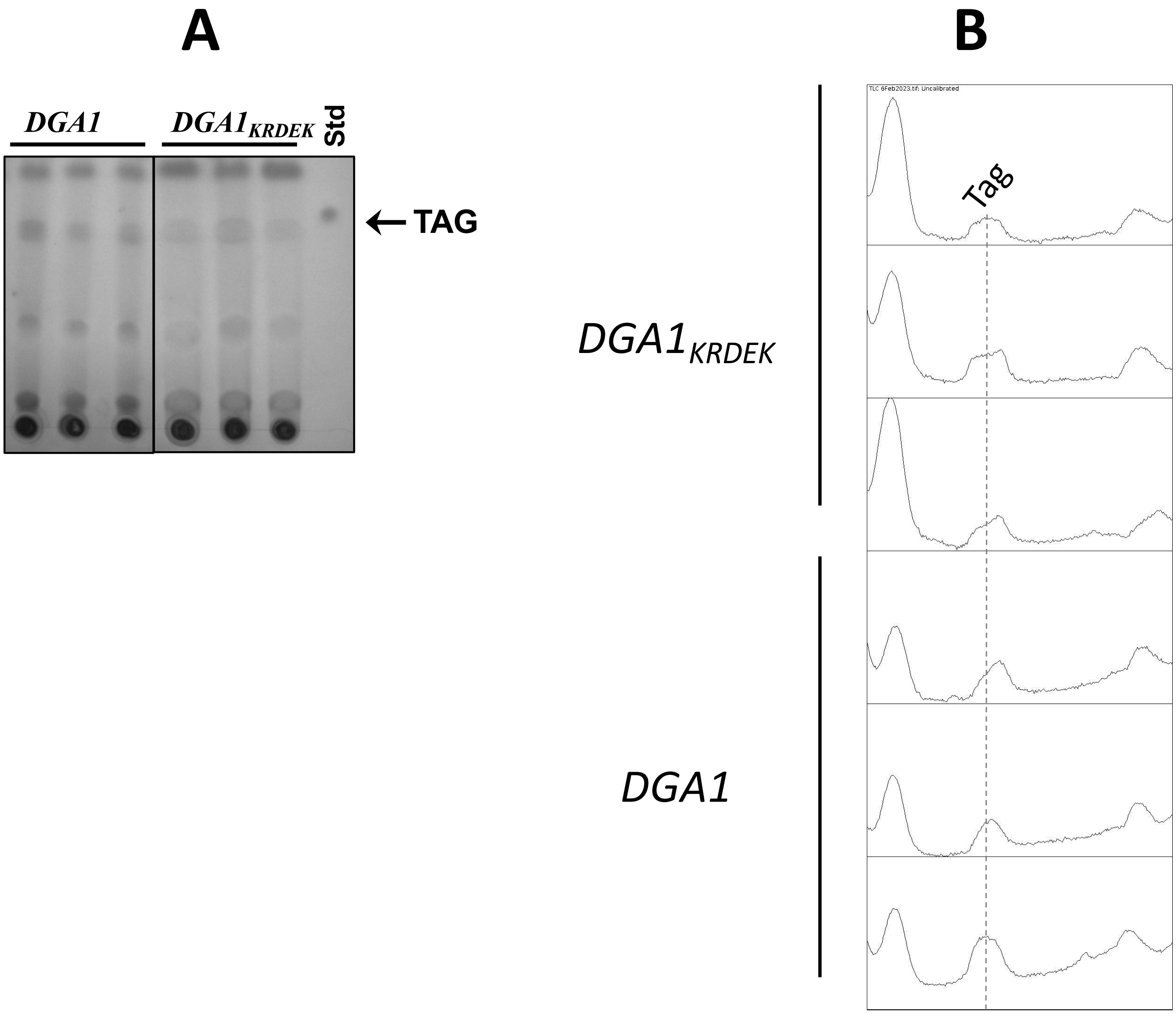

Figure S2. Lipid profile of are1 are2 lro1 DGA1 and are1 are2 lro1 DGA1$_{KRDEK}$ in log phase growth. A. Total yeast lipids were extracted from triplicate samples of the Indicated strains with methanol/chloroform. Lipid samples were resolved by TLC on silica gel plates and visualized by staining with Coomassie Blue. The position of TAG based on mobility of the tristearin standard (Std) and absence of a spot in *are1 are2 lro1 dga1* samples is indicated by the arrow. **B.** Densitometric analysis of the TLC plates and lanes plots produced by Image J.
